# *Streptomyces* produce a diphtheria toxin-like exotoxin that targets insects

**DOI:** 10.1038/s41564-026-02315-5

**Published:** 2026-04-30

**Authors:** Ying Xu, Reed M. Stubbendieck, Raghuvir Viswanatha, Ajda Krč, Lisa S. Baik, Won Se Suh, Yanhui Hu, Huan Wang, Linxiang Yin, Enzo Mameli, Anne van der Meij, John R. Carlson, Andrew C. Doxey, Pål Stenmark, Norbert Perrimon, Cameron R. Currie, Min Dong

**Affiliations:** 1 Department of Urology, Boston Children’s Hospital, Boston, MA USA; 2 Department of Surgery and Department of Microbiology, Harvard Medical School, Boston, MA USA; 3 State Key Laboratory of Animal Biodiversity Conservation and Integrated Pest Management, Institute of Zoology, Chinese Academy of Sciences, Beijing, China; 4 Department of Microbiology and Molecular Genetics, Oklahoma State University, Stillwater, OK USA; 5 Department of Bacteriology, University of Wisconsin-Madison, Madison, WI USA; 6 Department of Genetics, Blavatnik Institute, Harvard Medical School, Boston, MA USA; 7 Howard Hughes Medical Institute, Harvard Medical School, Boston, MA USA; 8 Department of Biochemistry and Biophysics, Stockholm University, Stockholm, Sweden; 9 Department of Molecular, Cellular and Developmental Biology, Yale University, New Haven, CT USA; 10 Department of Entomology and Nematology, University of California Davis, Davis, CA USA; 11 Department of Biochemistry and Biomedical Sciences, McMaster University, Hamilton, Ontario Canada; 12 Department of Biochemistry, University of Toronto, Toronto, Ontario Canada; 13 Department of Biology, University of Waterloo, Waterloo, Ontario Canada

**Keywords:** Bacterial toxins, Pathogens

## Abstract

*Streptomyces* and insects engage in complex interactions shaped by millions of years of evolution. While many beneficial relationships are well recognized, it remains unknown whether *Streptomyces* produce virulence factors targeting insects specifically. Here, through bioinformatic analysis, we identified diphtheria toxin (DT) homologues, which we named *Streptomyces* antiquus insecticidal proteins (SAIP), within a monophyletic lineage of *Streptomyces* that emerged more than 100 million years ago. SAIP is cytotoxic to insect cells and lethal to *Drosophila melanogaster*, suppressing neuronal activity and immune responses in vivo. Structural and functional studies validated that SAIP is homologous to DT and acts by ADP ribosylation of eukaryotic elongation factor 2. CRISPR–Cas9 screening identified the insect protein Flower as the SAIP receptor across a range of insects. Toxigenic *Streptomyces* can consume dead insects and produce bioactive secondary metabolites while growing on insect carcasses. These findings establish an insecticidal toxin in *Streptomyces* and demonstrate that *Streptomyces* have evolved highly specific virulence factors against insects.

## Abstract (web summary)

Bioinformatic, structural and functional analyses characterize *Streptomyces* antiquus insecticidal protein (SAIP) as a diphtheria toxin homologue that is lethal to insect cells and targets the Flower protein as a receptor

## Main

The genus *Streptomyces* is a paradigmatic example of the benefits of exploring the ecology and physiological diversity of microbes: antibiotics derived from *Streptomyces* have saved countless lives, and the metabolites they produce have served as anticancer, immunosuppressant and antiparasitic drugs or drug leads^1–3^. The amazing metabolic capacities of *Streptomyces* have been shaped by over 400 million years (Myr) of evolution as one of the most ubiquitous and diverse genera of terrestrial bacteria.

Over their long evolutionary history, *Streptomyces* have co-existed with insects^1,4–6^, and these bacteria are common components of insect microbiomes^7^. Recent work has detailed mutualistic relationships, with *Streptomyces* producing bioactive secondary metabolites to protect the food sources or larva of their insect hosts^8–11^. *Streptomyces* also play an important role in cellulose degradation for some insect herbivores^12^. Furthermore, some soil arthropods are known to be attracted by *Streptomyces* metabolites, and this attraction promotes dispersal of *Streptomyces* spores^13^. On the other hand, potential antagonistic interactions between *Streptomyces* and insects remain underexplored. It has been shown that some *Streptomyces* metabolites are toxic to insects and exposure to strains producing these molecules can kill insects^14^. However, the toxicity of these small-molecule metabolites is not specific to insects, and there are no known entomopathogenic *Streptomyces*.

Sophisticated protein exotoxins are often the major virulence factors in bacterial pathogens. Diphtheria toxin (DT) represents the first protein exotoxin identified and characterized^15^, and it is the sole virulence factor responsible for the disease diphtheria, a major cause of childhood death throughout human history^16^. DT comprises three domains: the N-terminal catalytic domain (C-domain), the middle membrane translocation domain (T-domain) and the C-terminal receptor-binding domain (R-domain)^17^. The C-domain is an ADP-ribosyltransferase, which modifies the conserved diphthamide residue in eukaryotic elongation factor 2 (eEF2), thus inhibiting protein translation^18–21^. As eEF2 is highly conserved, the species selectivity of DT is determined by its protein receptor, the heparin binding epidermal growth factor precursor (HB-EGF)^22,23^. For instance, DT cannot recognize mouse or rat HB-EGF due to several critical amino acid substitutions relative to the human version, rendering these species resistant to DT^23–25^.

The origin of DT remains a mystery. DT is encoded on a prophage and exhibits an altered GC content compared to the genome of its harbouring bacterium, *Corynebacterium diphtheriae*, suggesting that this toxin gene was acquired recently on evolutionary timescales^26^. Recent bioinformatic analyses reported eight DT-like proteins within various bacterial genera^27,28^. These DT-like proteins possess low sequence identity to DT but share conserved motifs, three-domain arrangement and overall protein length. Recent studies resolved the crystal structure of three of these DT-like proteins, named *Austwickia chelonae* toxin 1 (ACT1), *Seinonella peptonophila* toxin (PT) and *Streptomyces albireticuli* toxin (AT), confirming that they indeed share overall DT-like structures^28,29^. By constructing chimeric domain-swapped proteins between DT-like proteins and DT, it was shown that the putative C- and T-domains of these DT-like proteins are functional^28,29^. However, full-length DT-like proteins, without fusion to a DT-domain, have not been shown to be toxic to any cells or species.

Here we identified and characterized a family of DT-like proteins found in a monophyletic clade of *Streptomyces*. We found that these *Streptomyces* DT-like proteins are functional toxins recognizing insect cells and are toxic to insects. Using CRISPR–Cas9 mediated genome-wide screening, we identified an insect protein as the toxin receptor, thus establishing the insect-targeting specificity at the molecular and mechanistic level for this family of exotoxins in *Streptomyces*.

## Results

### *Streptomyces* harbours a protein homologous to DT

In an initial effort to understand the origin of DT, we searched for DT homologues in GenBank and manually selected 18 sequences (Supplementary Table 1) that contain (1) key signature motifs of DT; (2) three putative domains similar to DT; and (3) overall protein length similar to DT. All are from bacteria within the phylum Actinomycetota, including both sequences recently deposited into the database and previously reported DT-like proteins^27,28^. Most are isolated cases represented by a single deposited sequence in GenBank, sharing ~22–42% identity to DT and ~22–64% identity with each other (Supplementary Table 2). One exception is that there are eight proteins sharing 91–99% identity with each other, probably representing natural variants of the same protein (Supplementary Table 3). These eight variants are all found in *Streptomyces* (Supplementary Table 1) and have not been previously characterized.

**Fig. 1:**
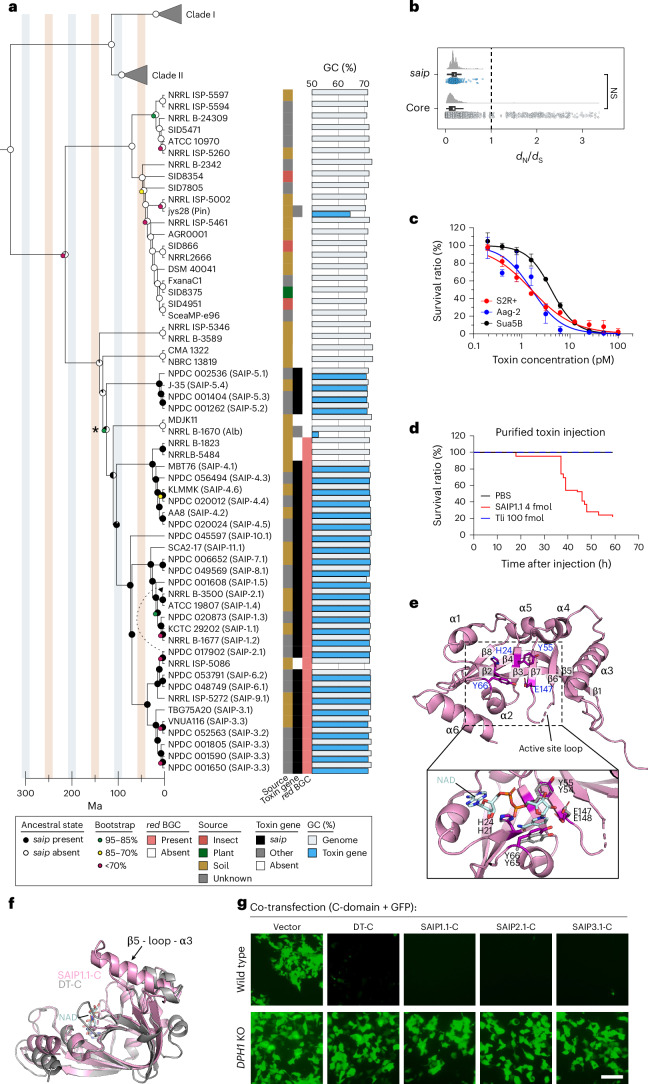
A clade of *Streptomyces* encodes a DT-like protein targeting insects. **a**, Subset of a phylogenetic tree built from 93 conserved single-copy bacterial genes (GenProp0799). The phylogeny was rooted on *Frankia alni* ACN14a (root not to scale, see Supplementary Fig. 1 for the full phylogenetic tree with all taxa). The branch lengths indicate estimated divergence times calculated on the basis of the split between *Kitasatospora* and *Streptomyces* (382 Ma, CI 250–514 Ma) and the split between the two major clades of *Streptomyces* (132 Ma, CI 82–177 Ma)^6^, which are represented as collapsed triangles and labelled Clade I and Clade II. Ancestral state reconstruction probabilities, calculated using Mesquite^68^, and bootstrap support are indicated at the nodes. A potential LGT event was determined through reconciliation with the *saip* gene phylogeny, and the directionality of transfer is indicated with a dashed and curved arrow. Bootstrap values >95% are not shown. The boxes to the right of the taxa indicate the strain source, the presence or absence of a DT homologue encoded in the genome, and the presence or absence of the *red* biosynthetic gene cluster (BGC, see Supplementary Fig. 6). The identity of the corresponding toxin is listed in parentheses next to the strain. The grey and blue bars on the right indicate the GC content of the genome and, if applicable, the toxin-encoding gene, respectively. Asterisk denotes the inferred ancestral node for the origin of SAIP. **b**, Raincloud plot showing pairwise *d*_N_/*d*_S_ ratios for *saip* and 93 conserved single-copy core genes. Each point represents a single comparison. All comparisons where either *d*_N_ or *d*_S_ were equal to 0 were filtered out of the dataset. The left and right bounds of the box plots indicate the 75th and 25th percentiles, respectively. The horizontal black bars indicate the medians. The whiskers extend from the bounds of the box to the largest and smallest values that are no further than 1.5× the interquartile range (IQR). A two-sided bootstrap test for the equality of means was used to compare the *d*_N_/*d*_S_ ratios of the *saip* genes to the core genes (replicates = 1,000). NS, not significant. **c**, Sua5B, Aag2 and S2R+ insect cells were exposed to SAIP1.1 for 4 days. Cell viabilities were quantified using MTT assays and data were collected from 3 independent replicates and plotted. Data are shown as mean ± s.d. **d**, *D. melanogaster* (Hml-gal4; UAS-GFP) were injected with SAIP1.1 (4 fmol), another DT-like protein Tli (100 fmol) or PBS. Survival curves are shown, *n* = 23. **e**, Crystal structure of the catalytic domain of SAIP1.1 (SAIP1.1-C) solved by molecular replacement and refined to 3.0 Å. The structure exhibits a typical ADP-ribosyltransferase (ADPRT) fold. The active site residues and the active site loop are labelled. The area marked with the rectangle is enlarged, with NAD (light blue) modelled into the active site and the key catalytic residues marked for both SAIP1.1-C (bold) and DT (PDB 1TOX). **f**, Superimposition of the catalytic domains of SAIP1.1 (pink) and DT (grey) (PDB 1TOX). **g**, Co-transfecting DT-C or SAIP1.1-C, SAIP2.1-C and SAIP3.1-C domains with GFP blocks GFP expression in wild-type HEK293T cells, but not in *DPH1* KO HEK293T cells. Representative pictures are from one of three independent experiments. Scale bar, 100 μm. Source data

**Extended Data Fig. 1:**
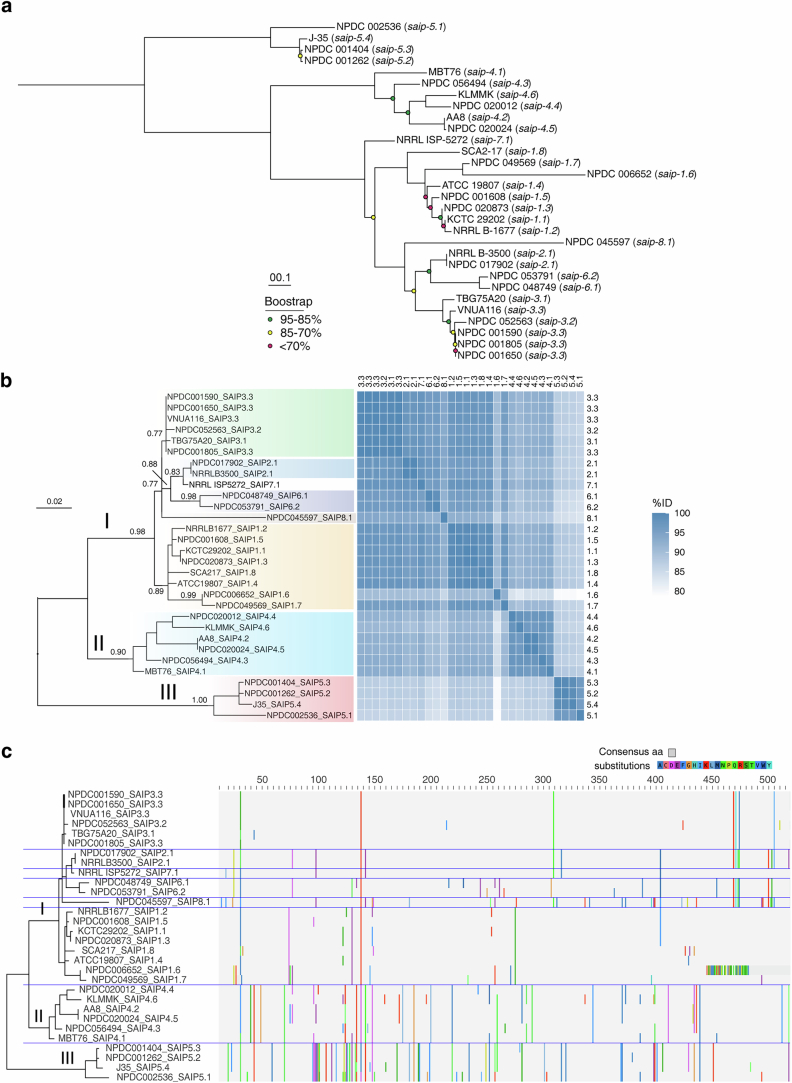
Phylogenetic analysis of *saip* genes and classification of SAIP subtypes. **(a)** Midpoint-rooted *saip* gene phylogeny. The *saip* alleles are indicated in parentheses. The phylogeny is drawn to scale, with branch lengths measured in the number of substitutions per site, as indicated by the scale bar. All positions containing gaps and missing data were eliminated (complete deletion option). This alignment is based on a total of 1,434 positions in the final dataset. **(b)** A heatmap of pairwise identities (right) was mapped on to the SAIP phylogeny (left). SAIP protein sequences can be broadly grouped into three major clusters (I, II, and III), and further subdivided phylogenetically into 8 subgroups. Toxin sequences were numbered based on their subgroup membership (for example, 1.1, 1.2, …, 2.1, 2.2, etc.). aLRT (approximate likelihood ratio test) branch support is shown above major clades in the tree. **(c)** The protein sequence alignment (right) was mapped onto the SAIP phylogeny, with consensus amino acids shown in gray and amino acid substitutions shown as colored lines. Distinct substitution profiles are evident for each subgroup indicative of their common ancestry and phylogenetic structure.

To assess the prevalence of this protein and its variants, we expanded the search to all publicly available *Streptomyces* genomes in GenBank, Joint Genome Institute Genome, and Natural Products Discovery Center databases. We further conducted genome sequencing of strains from our own *Streptomyces* strain collection and from the USDA-ARS culture collection (NRRL), selecting strains with >99% 16S rRNA identity to the strains in GenBank containing variants of this DT homologue. Through this large-scale effort, we identified an additional 22 *Streptomyces* strains harbouring this protein, with a total of 26 variants that can be grouped into 8 subtypes (Fig. 1a and Extended Data Fig. 1a–c, strain information in Source Data for Fig. 1, unique sequences are denoted numerically such as 1.1, with the first number indicating the subtype).

### DT-like protein is vertically inherited in a monophyletic clade

We next conducted genomic analyses of these *Streptomyces* strains to explore the evolutionary distribution of this DT homologue and to test for potential lateral gene transfer (LGT), a process that played a critical role in the evolution of DT in *Corynebacterium*^30^. First, we constructed a core-genome phylogeny from 93 conserved single-copy bacterial genes for all 30 strains containing the protein, 9 closely related strains that do not encode this DT-like protein and 72 representatives of other *Streptomyces* lineages (Fig. 1a and Supplementary Fig. 1). Compared to the major *Streptomyces* lineages, the lineage containing the DT homologue has been undersampled and there are few sequenced representatives available in public databases. The *Streptomyces* strains containing this DT homologue form a monophyletic lineage (Fig. 1a), with 30 of the 35 strains within this lineage possessing this DT homologue (Fig. 1a).

There is no difference in the GC content of this DT homologue gene (mean = 71.8%, s.d. = 0.48%) compared to the GC content of the whole genome (mean = 72.3%, s.d. = 0.44%) (Fig. 1a), and DNA *Z*-curve segmentation analysis detected no genome segmentation near the gene encoding this DT-like protein in these *Streptomyces* strains (Supplementary Fig. 2), indicating that there are no LGT events near this locus. We further found that the neighbouring genes have a high degree of synteny across strains, with differences in gene order and composition congruent with the *Streptomyces* phylogeny (Supplementary Fig. 3). Finally, we determined that the ratio of non-synonymous to synonymous substitutions (*d*_N_/*d*_S_) for this DT-like protein encoding gene (mean *d*_N_/*d*_S_ = 0.20) was not significantly different from the *d*_N_/*d*_S_ ratio of all pairwise combinations of the 93 core genes used to construct the core-genome phylogeny (mean *d*_N_/*d*_S_ = 0.17) (Fig. 1b), suggesting that this protein is under strong stabilizing selection.

To further evaluate the evolutionary origin of this DT homologue, we estimated divergence times using the split between *Streptomyces* and *Kitasatospora* and the split between the two major clades of *Streptomyces* as calibration points^6^. These analyses support that the toxigenic clade of *Streptomyces* arose ~125 million years ago (Ma, confidence interval (CI) 95–156 Ma) and before the split between the two major clades of *Streptomyces*. Our phylogenetic analyses support this vertical inheritance, with one probable LGT event of the allele from strain NPDC 017902 into strain NRRL B-3500, and two or three putative losses (Fig. 1a and Extended Data Fig. 1a). In conclusion, these findings suggest that there was a single origin of this DT-like protein in a monophyletic clade of *Streptomyces*, and it has been maintained vertically for over 100 Myr under strong stabilizing selection.

### *Streptomyces* DT-like protein is toxic to insects

**Extended Data Fig. 2:**
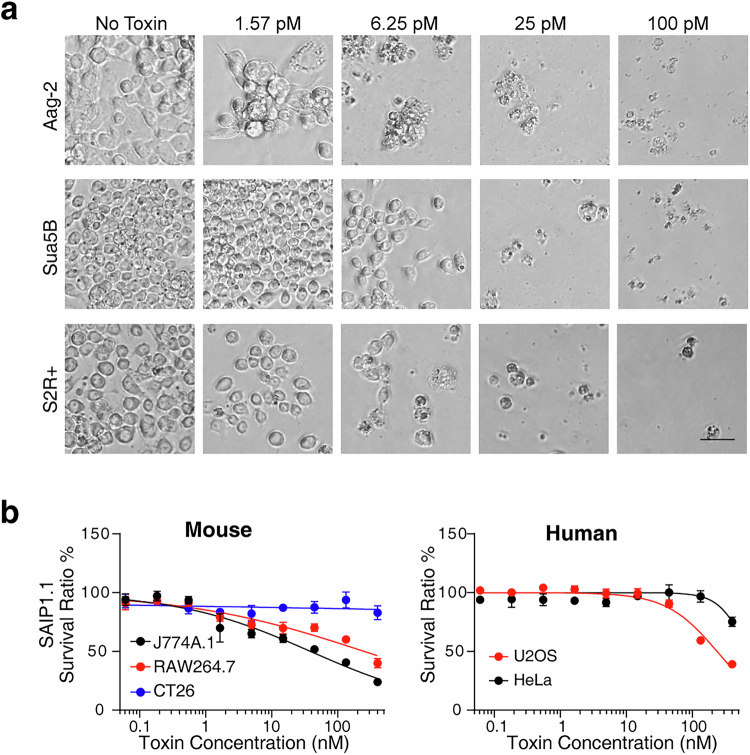
SAIP1.1 is highly toxic to insect cells. **(a-b)** Sua5B, Aag2, and S2R+ cells were exposed to SAIP1.1 for 4 days. Representative images were from one of three independent experiments and shown in **(a)**. Besides three insect cell lines, various mouse and human cells were also tested through MTT cell viability assays in parallel. Their sensitivities to SAIP1.1 was quantified and plotted **(b)**. Data are shown as mean ± s.d., n = 3 (three independent biological replicates), scale bar, 25 μm.

**Extended Data Fig. 3:**
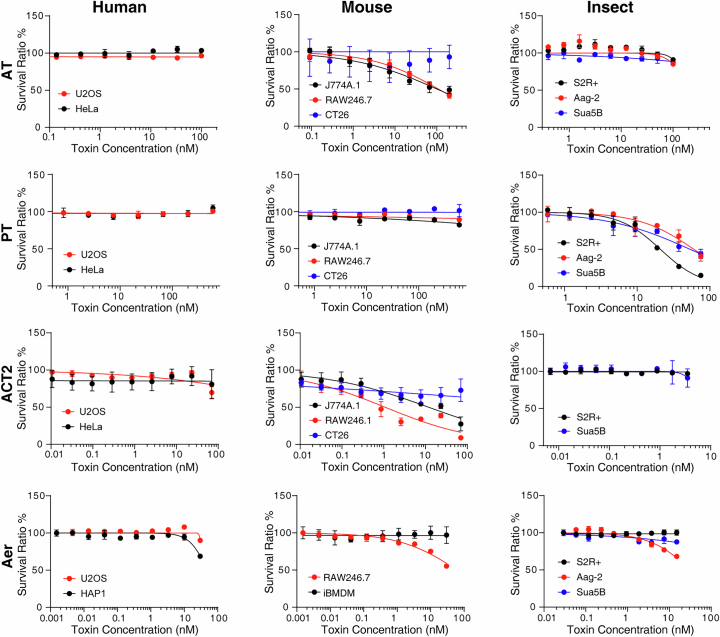
DT-like proteins AT, PT, ACT2, and Aer showed no cytotoxicity to human, mouse, or insect cells. A range of cell lines were exposed to a series of concentrations of toxins for 4 days and cell viability was measured using MTT assays. The percentage of survival cells was quantified and plotted. Data are shown as mean ± s.d., n = 3 (three independent biological replicates).

We next recombinantly produced a representative protein (sequence 1.1) in *Escherichia coli* and evaluated its toxicity on cultured cell lines from human (U2OS and HeLa), mouse (CT26, J774A.1 and RAW264.7) or insects (*Drosophila melanogaster*: S2R+, *Aedes aegypti*: Aag2 and *Anopheles coluzzii*: Sua5B). This protein induced death in three insect cell lines, with toxin concentrations resulting in death of 50% cells (IC_50_) below 10 pM (Fig. 1c). By contrast, there was minimal toxicity on mouse and human cells until doses exceeded 10 nM (Extended Data Fig. 2). In parallel, we also evaluated three previously reported DT-like proteins (AT, PT and ACT2), and none exhibited any toxicity on insect, mouse or human cells (Extended Data Fig. 3). We further tested another DT-like protein found in our search (from *Klebsiella aerogenes*, referred to here as Aer, Supplementary Table 1), and it showed no toxicity to any cells tested (Extended Data Fig. 3).

We next evaluated in vivo toxicity to insects by micro-injection of this *Streptomyces* DT-like protein into the haemocoel of *D. melanogaster*, and found that injection of 4 fmol killed ~80% of flies within 60 h (Fig. 1d). As controls, injection of a different DT-like protein (designed Tli, Supplementary Table 1) showed no toxicity (Fig. 1d). These findings suggest that this *Streptomyces* DT-like protein, maintained vertically for over 100 Myr, specifically targets insects. We thus name it *Streptomyces* antiquus insecticidal protein (SAIP).

### SAIP contains an ADP-ribosyltransferase domain that inhibits eEF2

SAIP shares ~27% sequence identity to DT (Supplementary Table 3) and has many sequence signatures suggesting that it is homologous to DT, including conserved residues within the N-terminal region consistent with ADP-ribosyltransferase activity, and a pair of Cys residues located between the N-terminal putative catalytic domain (C-domain) and the putative membrane translocation domain (T-domain), which probably form the disulfide bond connecting two domains.

To determine the molecular basis for the toxicity of SAIP, we solved the X-ray crystal structure of a SAIP C-domain to 3.0 Å resolution (Fig. 1e,f and Supplementary Table 4). The structure revealed typical features of an ADP-ribosyltransferase, with a split β-sheet (β1-3-5-6-7 and β2-4-8) and several α-helices surrounding the β-sheet^19^. The active site loop between β6 and β7 contains the catalytic Glu147, as well as a Gln145 upstream, forming the Q-X-E motif that is important for ribosyltransferase activity^31,32^. The key NAD-binding and catalytic residues are conserved between SAIP and DT (for example, Tyr55, Glu147, His24 and Tyr66), and the loops surrounding the active site are positioned in similar conformations^17,31^ (Fig. 1e). According to Foldseek structure search^33^, the overall structure is most similar to the DT C-domain (PDB 1DTP, TM-score = 0.736, RMSD = 3.32 Å, calculated on the basis of Cα atoms of aligned residues) (Fig. 1f).

**Extended Data Fig. 4:**
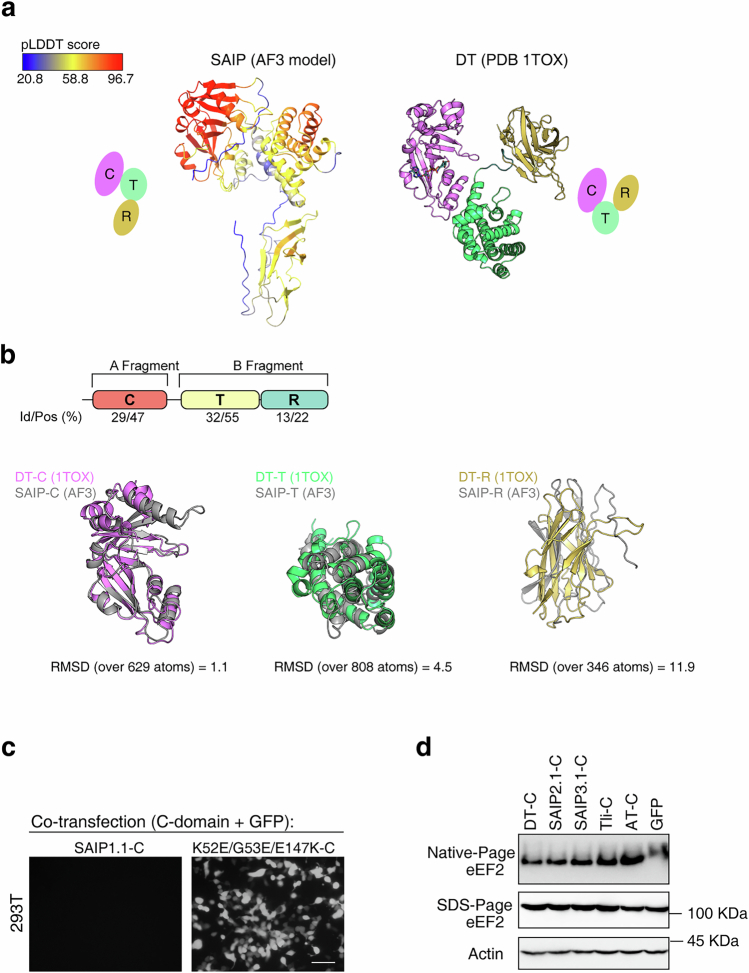
SAIP is homologous to DT and targets eEF2. **(a)** AlphFold3 prediction of SAIP structure (right panel), showing three-domain structure similar to DT (left panel). SAIP model is colored by pLDDT score, showing confidence of the prediction (higher scores indicate higher confidence and usually better accuracy of the prediction). DT model (PDB 1TOX) is colored by domains. The schematic of the domain organization is shown on the side of each model (C: catalytic domain, T: translocation domain, R: receptor-binding domain). **(b)** Upper panel: cartoon illustration of the three-domain arrangement of SAIP, with the sequence identity (Id%) and similarity (Pos%) compared to DT listed for each domain. Lower panel: Domain-by-domain superposition of SAIP Alphafold3 model and DT model (PDB 1TOX). DT C, T and R domains are colored in violet, green and yellow respectively, and SAIP domains are colored in grey. RMSD scores were calculated after alignment in PyMOL. **(c)** Co-transfection of SAIP1.1-C domain with GFP can block the expression of GFP in HEK293T, and this suppression is abolished when three conserved residues in the C domain were mutated. Representative pictures were from one of three independent experiments. Scale bar = 100 μm. **(d)** Cell lysates from DT-C domain, different DT orthologs-C domains or GFP transfected HEK293T cells were separated by native PAGE or SDS/PAGE and analyzed by western blotting using the anti-eEF2 741-858aa antibody. Representative images were from one of three independent experiments. Source data

Superimposing the structure of the SAIP1.1 C-domain to the DT C-domain demonstrates major differences in the β5-loop-α3 motif that faces the R-domain in the full-length DT structure (Fig. 1f). Both the loop and adjacent α-helix are longer in SAIP1.1-C and therefore clash with the R-domain of DT, suggesting that SAIP might exhibit somewhat different overall domain organization compared to DT. This hypothesis is in agreement with the AlphaFold3 prediction of the full-length toxin (Extended Data Fig. 4a). Individual domains still show fold agreement between DT and SAIP, with the C-domain being the most similar and the R-domain the least similar in fold (RMSD = 11.9) (Extended Data Fig. 4b).

DT is known to target eEF2 through ADP ribosylation of a conserved diphthamide residue, resulting in inhibition of protein synthesis^19^. This diphthamide is a post-translationally modified histidine, which is conserved in eEF2 but not in bacterial EF2. Thus, DT is not toxic to bacteria^20^. This modification can be blocked by disrupting genes involved in diphthamide biosynthesis, such as diphthamide biosynthesis 1 (*DPH1*)^20,34^. As expected, expression of DT C-domain in HEK293 cells suppressed translation of green fluorescent protein (GFP) from a co-transfected vector but did not affect GFP expression in *DPH1* knockout (KO) cells (Fig. 1g). We tested C-domains from SAIP1.1, 2.1 and 3.1, and all inhibited GFP expression in HEK293 cells but not in *DPH1* KO cells (Fig. 1g). Furthermore, mutating three residues in the SAIP C-domain (K52E/G53E/E147K) corresponding to the conserved residues crucial for DT catalytic activity abolished the inhibition of GFP expression in HEK293 cells^35,36^ (Extended Data Fig. 4c). It was previously shown that eEF2 modified by DT migrates faster on a native polyacrylamide gel than unmodified eEF2 (ref. ^34^). Consistently, eEF2 from cells that express DT C-domain or SAIP C-domain migrated faster on native gels than eEF2 from control cells (Extended Data Fig. 4d). Together, these findings indicate that SAIP shares the same mode of action as DT: inhibiting protein synthesis via ADP ribosylation of diphthamide in eEF2.

### Identification of an insect protein receptor for SAIP

**Fig. 2:**
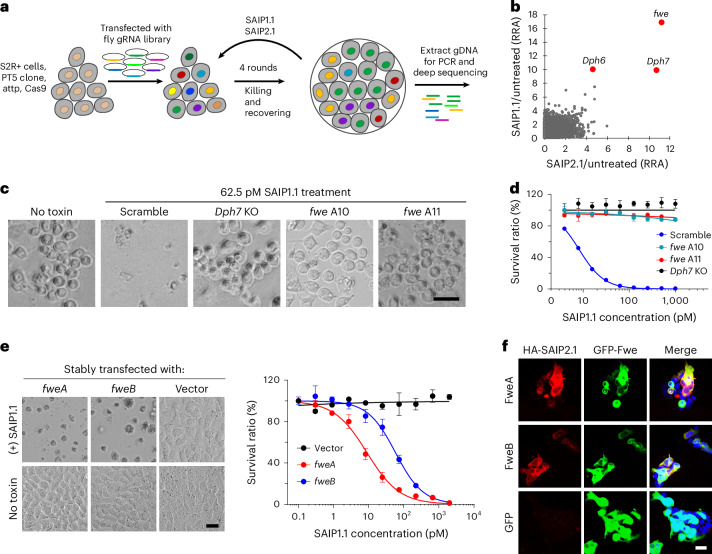
Fwe is the insect protein receptor for SAIP. **a**, Schematic of the pooled screen using SAIP1.1 or SAIP2.1 toxin in PT5/S2R+ cells. **b**, Top hits from the two screens. Genes identified were ranked on the basis of the robust rank aggregation (RRA) score. **c**,**d**, Mixed stable KO cells lacking *Dph7* (*Dph7* KO), two *fwe* KO single clones (*fwe* A10 and *fwe* A11) and a control S2 cell line expressing scramble sgRNAs (Scramble) were exposed to a series of concentrations of SAIP1.1 for 4 days. **c**, Representative images with 62.5 pM SAIP1.1 treatment are from 1 of 3 independent experiments. **d**, Cell viability was measured using MTT assays, and data were collected from 3 independent replicates and plotted. **e**, Sensitivity to SAIP1.1 of various HeLa cell lines expressing *Drosophila fwe* isoforms (*fweA* or *fweB*) or blank vector were assessed. Representative images with SAIP1.1 (2 nM, 4 days) treatment are from 1 of 3 independent experiments. **f**, Ectopic expression of Fwe–GFP in HeLa cells via transient transfection enhanced the binding of HA-tagged SAIP2.1 (red) to GFP-positive cells (green). Nuclei were labelled with Hoechst dye (blue). Representative images are from 1 of 3 independent experiments. Data are shown as mean ± s.d., *n* = 3. Scale bar, 25 μm.

As eEF2 is highly conserved, the species specificity of SAIP can be determined by its receptor. We previously established a genome-wide CRISPR–Cas9 mediated screening method in *D. melanogaster* S2 cells^37–39^. Using this approach, we carried out two independent genome-wide screens using SAIP1.1 and 2.1 (Fig. 2a). The top hit from both screens is *fwe*, which encodes a protein named Flower (Fwe) (Fig. 2b and Supplementary Table 5). Other top hits include *Dph7* (CG3184) and *Dph6* (CG1578), both involved in diphthamide synthesis.

**Extended Data Fig. 5:**
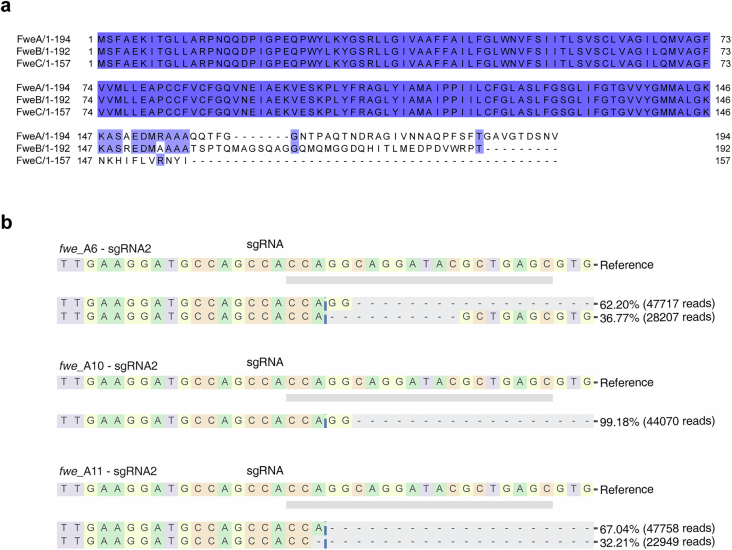
Schematic description of different *fwe KO* S2R+ clones. **(a)** Protein sequence alignment for three Fwe splicing forms. **(b)** Deep sequencing results of different *fwe KO* S2R+ clones were plotted and quantified based on mutation ratios. *fwe* A10 and *fwe* A11 were utilized in Fig. 2c, and *fwe* A6 was utilized in Extended Data Fig. 9.

Fwe is a small membrane protein first identified in *D. melanogaster*^40,41^, with orthologues found throughout the kingdom Metazoa. Fwe has three splice variants in *D. melanogaster*, termed A, B and C (also known as Ubi, Lose-A and Lose-B), which share residues 1–147 (full-length FweA has 194 residues) but possess different C termini^40,41^ (Extended Data Fig. 5a). The *D. melanogaster* Fwe was shown to consist of three transmembrane domains, with its C-terminal region experimentally demonstrated to be outside of the cell surface^41^. It has been demonstrated that differential levels of Fwe splice variants on neighbouring cells can mediate win/lose decisions in cell competition during development and aging^41–43^. Fwe is also proposed to have Ca^2+^ channel activity^40,44,45^. The mammalian orthologue of Fwe (CACFD1 protein) has been reported to have a role in granule endocytosis in immune cells and in tumour development^46–48^.

**Extended Data Fig. 6:**
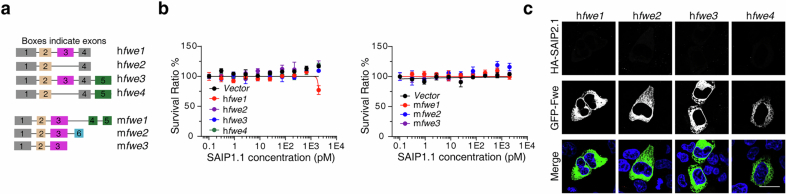
Human and mouse Fwe orthologs cannot function as receptors for SAIP. **(a)** Schematic of the different human and mouse *fwe* slicing isoforms. **(b)** HeLa cells stably overexpressed human *fwe* (CACFD)*-1*, *2*, *3*, *4* or mouse *fwe* (mCACFD)*-1*, *2*, *3* were exposed to a series of concentrations of SAIP1.1 for 4 days and cell viability was measured using MTT assays. The sensitivities to SAIP1.1 were quantified and plotted. **(c)** Human Fwe cannot enhance the binding of HA-tagged SAIP2.1 to cells. HeLa cells transiently expressing different human Fwe-GFP fusion proteins were exposed to 50 nM HA-SAIP2.1 for 5 h, and subjected to immunostaining of HA (Red). Nuclei were labeled with Hoechst dye (Blue). The GFP signal was recorded directly under a microscope (Green). Representative images were from one of three independent experiments. Data are shown as mean ± s.d., n = 3 (Three independent biological replicates), scale bar = 25 μm.

We generated two independent *fwe* KO S2 cell clones (Extended Data Fig. 5b). Both lines became highly resistant to SAIP (Fig. 2c,d), similar to *Dph7* KO S2 cells (Fig. 2c,d). Human cell lines, such as HeLa cells, are resistant to SAIP (Fig. 2e), and two HeLa cell lines that stably express either *D. melanogaster* FweA or FweB became highly sensitive to SAIP, exhibiting IC_50_ values of ~9 pM and 58 pM, respectively (Fig. 2e). Furthermore, expression of GFP-tagged *D. melanogaster* FweA or FweB in HEK293T cells mediated binding of SAIP to transfected cells (Fig. 2f). By contrast, HeLa cells transfected with human or mouse *Fwe* orthologues (encoded by *CACFD1* or *Cacfd1* genes) remain resistant to SAIP (Extended Data Fig. 6a,b), and overexpression of human Fwe in HeLa cells did not mediate binding of SAIP (Extended Data Fig. 6c).

### SAIP recognizes a broad range of insect Fwe

**Fig. 3:**
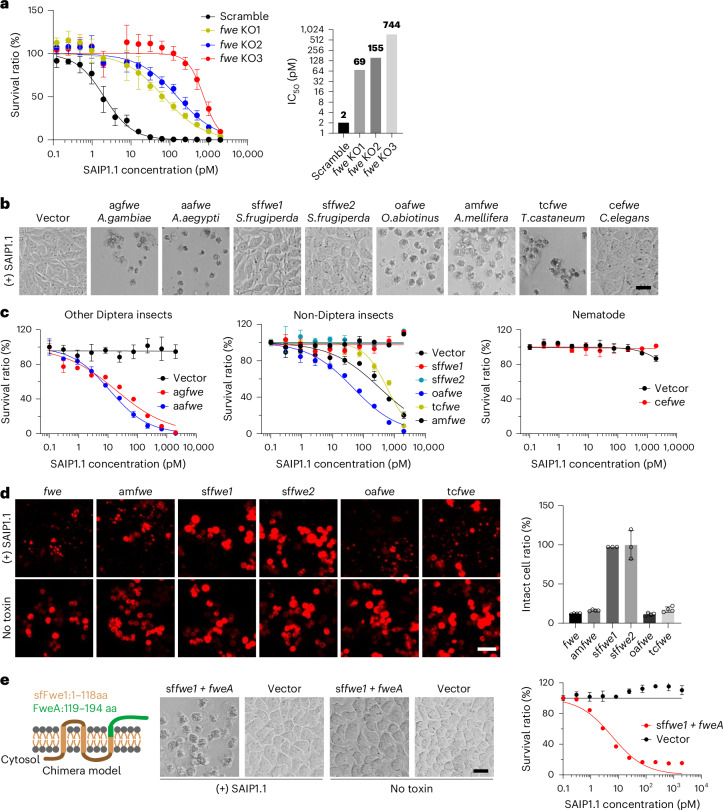
SAIP recognizes a broad range of insect Fwe. **a**, Three single gRNAs targeting *Anopheles fwe* (ag*fwe*) and one gRNA targeting scramble sequence were transfected into Sua5B/Cas9 cells to generate stable knockout cell lines. Cell viability was measured using MTT assays after 4-day SAIP1.1 treatment. The toxin concentration resulting in 50% cell death was defined as IC_50_ (plotted on the left). **b**,**c**, The indicated *fwe* orthologues were stably expressed in HeLa cells. Cells were exposed to SAIP1.1 with a series of concentrations for 4 days. Representative images from the 2 nM group are shown in **b**. Sensitivities were quantified and plotted in **c**. **d**, *Drosophila fwe* and the indicated *fwe* orthologues, all fused with N-terminal mCherry and C-terminal VHH05 tags, were expressed in *fwe* KO S2R+ cells via transient transfection and then exposed to SAIP1.1 (250 pM) for 48 h. Representative images are from one of three independent experiments (left). Percentages of cells remaining intact were recorded and plotted (right). **e**, HeLa cells stably expressing a chimera *Spodoptera frugiperda fwe1* with *Drosophila fweA* C-terminal (sf*fwe1+fweA*) or blank vector were treated with a titration of SAIP1.1 for 4 days, and sensitivity to SAIP1.1 was quantified via MTT assay. Representative pictures (2 nM SAIP1.1) are from 1 of 3 independent experiments. Data are shown as mean ± s.d., *n* = 3 independent biological replicates. Scale bar, 25 μm.

We next examined whether SAIP could recognize *fwe* orthologues in additional insect species. Mosquitos such as *A. aegypti* and *Anopheles gambiae* complex are major disease vectors. We generated three *fwe* KO *A. coluzzii* Sua5B cells using the CRISPR–Cas9 platform previously developed^49^, and found that all KO cells exhibited reduced sensitivity to SAIP (Fig. 3a). Consistently, exogenous expression of *fwe* orthologues from *A. aegypti* (aa*fwe*) or *A. gambiae* (ag*fwe*) rendered HeLa cells sensitive to SAIP, with an IC_50_ value of ~20 pM (Fig. 3b,c).

Both mosquitos and *D. melanogaster* belong to the order Diptera. We further tested *fwe* from other representative insects: wasp (*Orussus abietinus*, oa*fwe*, Hymenoptera), honeybee (*Apis mellifera*, am*fwe*, Hymenoptera), beetle (*Tribolium castaneum*, tc*fwe*, Coleoptera) and moth (fall armyworm, *Spodoptera frugiperda*, with two forms: sf*fwe1* and sf*fwe2*, Lepidoptera). In addition, we also tested *Fwe* from nematode (*Caenorhabditis elegans*, ce*fwe*). Expression of *fwe* orthologues from wasp, honeybee or beetle all sensitized HeLa cells to SAIP (Fig. 3b,c). By contrast, the two moth forms and the nematode form did not confer sensitivity to SAIP in HeLa cells (Fig. 3b,c). To confirm these findings, we further expressed these insect *fwe* orthologues in *fwe* S2 KO cells (Fig. 3d), and expression of wasp, honeybee and beetle Fwe restored sensitivity of KO cells to SAIP, but moth and nematode Fwe did not (Fig. 3d).

**Extended Data Fig. 7:**
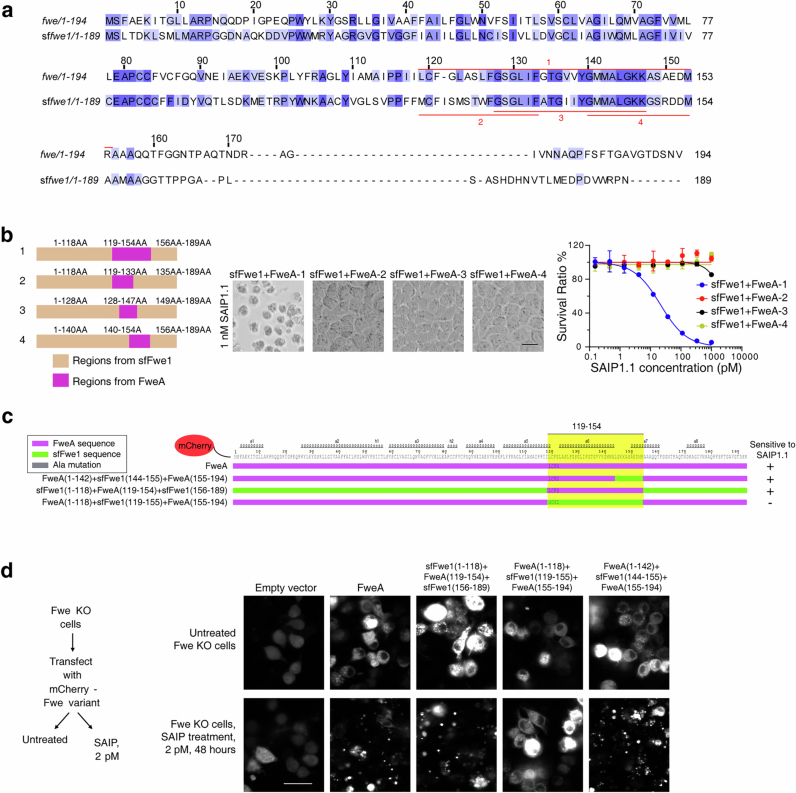
Mapping the key region in Fwe for SAIP recognition. **(a)** Sequence alignment between FweA and sfFwe1. **(b)** Chimaera sfFwe1+FweA-1/2/3/4 (The corresponding region of *Spodoptera frugiperda* Fwe1 was replaced with different regions between amino acids 119 and 154 of *Drosophila* FweA) or blank vector were stably expressed in HeLa cells respectively, and then treated with a titration of SAIP1.1 for 4 days. Each replaced regions were marked out by purple boxes in the right schematic of chimaera Fwes. sFwe1+FweA-1 is the only one that can sensitize HeLa cells to SAIP1.1 treatment. Their sensitivities to SAIP1.1 were quantified via MTT assays. Representative pictures (1 nM SAIP1.1) were from one of three independent experiments. **(c)** Schematic of the different chimaera proteins of sfFwe1+FweA. All proteins were fused with mCherry tag on their N-terminus. **(d)** Chimaera sfFwe1+FweA proteins and *Drosophila* Fwe (FweA) were transiently expressed in *fwe KO* S2 cells respectively, and then treated with 2 pM SAIP1.1 for 48 h. Cells susceptible to SAIP died and become debris, whereas cells resistant to SAIP remain intact and can be visualized by their expression of mCherry. The presence of mCherry-positive cell debris following SAIP treatment indicates that the transfected Fwe-mCherry protein has restored sensitivity to SAIP. Representative pictures were from one of three independent experiments. Data are shown as mean ± s.d., n = 3, scale bar = 25μm.

We next mapped the region critical for SAIP recognition by swapping residues between *D. melanogaster* Fwe and sfFwe1 (Fig. 3e and Extended Data Fig. 7). Replacing the C-terminal region (residues 119–194) in sfFwe1 with the corresponding region of *D. melanogaster* Fwe resulted in a chimeric Fwe that sensitized HeLa cells to SAIP (Fig. 3e). Shortening the swapped region to residues 119–154 also restored the sensitivity to SAIP (Extended Data Fig. 7a,b), but further shortening to 119–133, 128–147 or 140–154 did not confer sensitivity to SAIP in HeLa cells (Extended Data Fig. 7b). We further validated these findings in *fwe* KO S2 cells: transfection of a chimeric sfFwe1 with the region 119–154 derived from fly Fwe restored sensitivity to SAIP (Extended Data Fig. 7c,d), whereas cells transfected with a reciprocal chimera remain resistant to SAIP (Extended Data Fig. 7c,d). A chimera of *Drosophila* Fwe containing residues changed to the aligned residues found in sfFwe1 within the region 144–154 restored sensitivity of *fwe* KO S2 cells to SAIP, but another chimeric Fwe containing more residue swaps (to sfFwe1 residues) across 119–154 did not mediate SAIP toxicity (Extended Data Fig. 7c,d). Together these findings suggest that residues 119–154 in the membrane proximal region are crucial for SAIP recognition, and residue variations within this region determine species susceptibility to SAIP.

### SAIP suppresses neuronal activity and immune responses in vivo

**Fig. 4:**
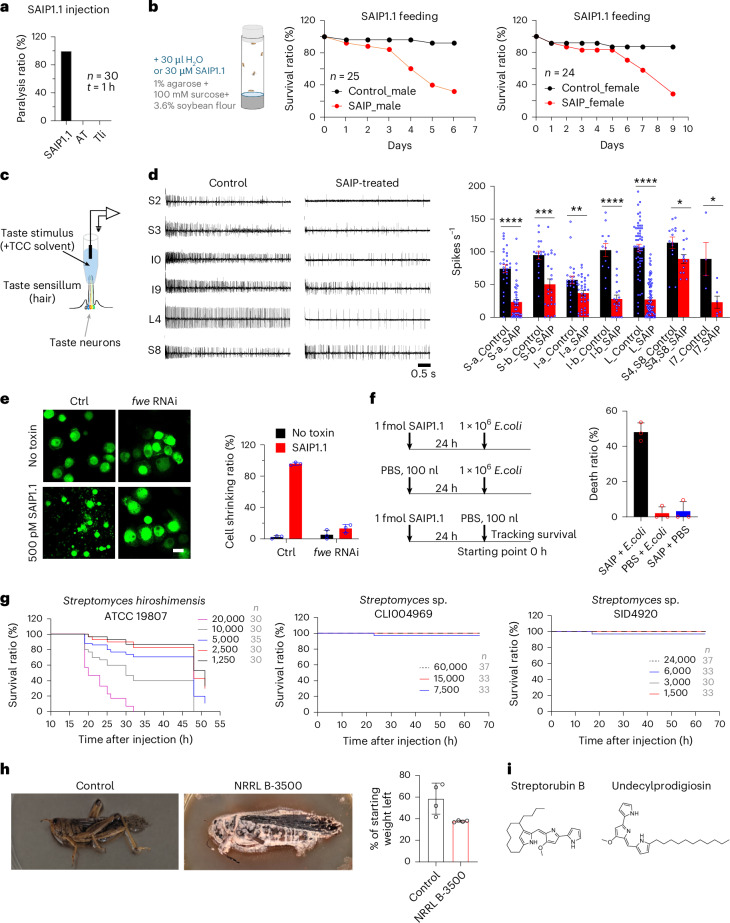
SAIP is toxic to *D. melanogaster* in vivo and the toxigenic *Streptomyces* can consume insect carcasses. **a**, SAIP1.1, AT or Tli (all at 100 fmol) were injected into 6–10-day-old *D. melanogaster* (Hml-gal4; UAS-GFP, male:female = 1:1). Flies developed paralysis after SAIP1.1 injection (1 h after injection). Thirty flies were injected for each toxin. **b**, Male and female *D. melanogaster* (strain w1118) were exposed to SAIP1.1 (30 µM SAIP1.1) or sterile water applied to the surface of an agarose food vial. Flies were moved to new vials daily, with SAIP1.1 or water added to the fresh vials each time. The mortality of the flies in each vial was recorded daily and plotted. **c**, Schematic of recordings from individual taste sensilla (adapted from ref. ^80^. **d**, Flies were exposed to SAIP (100 mM sucrose mixed with 20 µM SAIP1.1) for 7–8 days, and responses of their labellar taste sensilla to 100 mM sucrose were recorded. Responses were from 4–7 flies. Example traces of labellar taste neuronal responses to sucrose are shown (left side: control; right side: exposed to SAIP), and spike frequencies are quantified in the right panel (black, control; red, SAIP treated; NS, no significant change; ****P < 0.0001; ***P = 0.0003; **P = 0.005; *P = 0.0369 (S4, S8); *P = 0.0212 (I7); unpaired *t*-test; error bars are s.e.m. **e**, Haemocytes from Hml-gal4; UAS-GFP or Hml-gal4; UAS-GFP/UAS-fwe shRNA (v39596) *D. melanogaster* were exposed to SAIP1.1 (500 pM, 24 h). Representative images are from 1 of 3 independent experiments and percentages of shrinking GFP^+^ cells are shown. Data are shown as mean ± s.d. Scale bar, 10 μm. Ctrl, control. **f**, *D. melanogaster* (Hml-gal4; UAS-GFP) were pre-injected with PBS or SAIP1.1 (100 nl, 1 fmol). After 24 h, flies were then injected with 1 × 10^6^ *E. coli* (DH5a) or PBS. Survival ratios were quantified 24 h after injection of bacteria. Three independent experiments were done. Fly numbers for each experiment are listed in Source Data. Data are shown as mean ± s.d. **g**, The spores of a toxigenic *Streptomyces* (ATCC 19807) and two control strains were collected and injected into *D. melanogaster*. Survival rates were recorded and plotted. Fly numbers are shown in grey. **h**, SAIP^+^ strain *S. baldaccii* NRRL B-3500 exhibits robust growth on the exoskeleton of grasshopper cadavers, with an accumulation of secreted red-pigmented metabolites that includes the natural products undecylprodigiosin and streptorubin B (Extended Data Fig. 10). Images show growth 5 days after spore inoculation or water inoculation on two separate carcasses. Percentages of starting weight left were quantified and plotted as a bar graph (right). Data from four biological replicates. Data are shown as mean ± s.d. **i**, Mass spectrometry analysis of extracts from insect cadaver identified the secondary metabolites undecylprodigiosin and streptorubin B, which are known to have red-pigmented chemistry and antimicrobial activity. Source data

Analysis of single-cell RNA-sequencing data from the Fly Cell Atlas (FCA)^50^ revealed that the *fwe* gene is widely expressed, with sensory and motor neurons expressing higher levels than other cell types (Supplementary Fig. 4). The highest *fwe* expression is localized to multidendritic neurons, which are sensory neurons that coordinate muscle contraction (Supplementary Fig. 4). Consistent with these data, micro-injection of 100 fmol SAIP into *D. melanogaster* resulted in paralysis of all flies within 1 h (for example, flies failed to move/climb after tapping the culture tube) (Fig. 4a). As controls, two other DT-like proteins, AT and Tli, did not show any effect when injected into *D. melanogaster* (Fig. 4a).

We next explored whether it is possible for flies to become intoxicated through feeding on SAIP protein. High doses of SAIP were added and absorbed daily to the surface of an agarose food vial in which *D. melanogaster* was cultured, and we found that adding SAIP to the fly food in this manner resulted in gradual paralysis and eventual death of flies within several days (Fig. 4b and Supplementary Videos 1 and 2).

**Extended Data Fig. 8:**
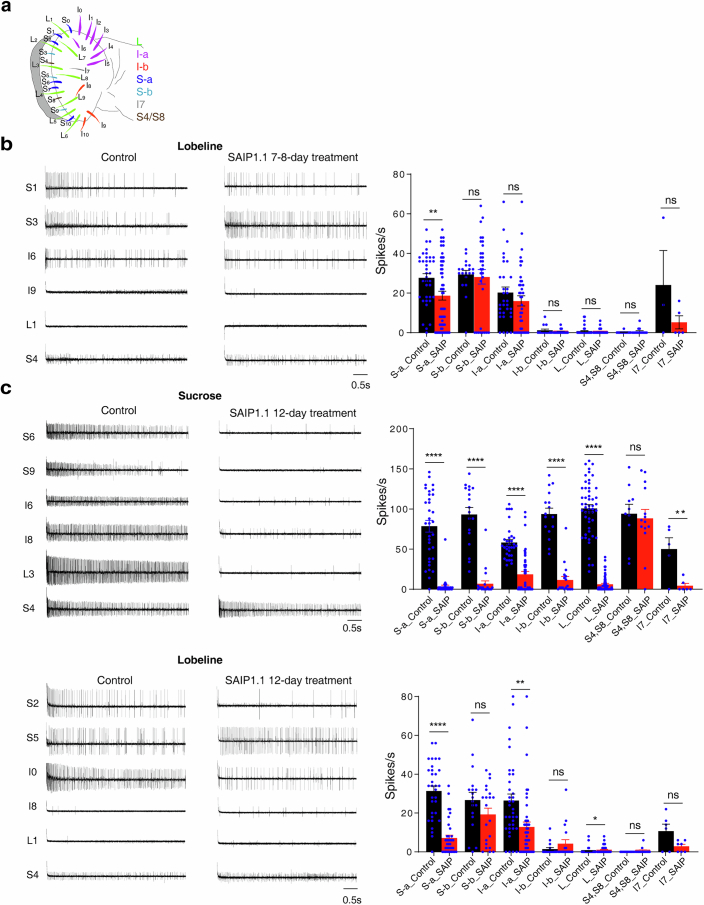
SAIP suppresses taste responses in vivo. **(a)** Schematic of fly labellar taste sensilla. **(b)** Flies were exposed to SAIP (raised in 100 mM sucrose containing 20 μM SAIP1.1) for 7-8 days and their labellar taste responses to 1 mM lobeline (a bitter compound) were recorded. Responses were from 4 ~ 7 flies. Representative traces of labellar taste neuronal responses are shown (left side: control; right side: exposed to SAIP) and corresponding spikes frequencies are quantified in the right panel (black: control; red: SAIP treated; *ns*=no significant change;**p = 0.0075 (S-a); unpaired t test; error bars are s.e.m.). **(c)** Flies were exposed to SAIP (raised in 100 mM sucrose containing 20 μM SAIP1.1) for 12 days, and their labellar taste responses to sucrose (upper panel) or lobeline (lower panel) were recorded. Representative traces of labellar taste neuronal responses are shown (left side: control; right side: exposed to SAIP) and corresponding spike frequencies are quantified in the right panel (black: control; red: SAIP treated; *ns*=no significant change; ****p < 0.0001; **p = 0.007 (Sucrose-I7);*p = 0.0394 (Lobeline-L); **p = 0.0016 (Lobeline-Ia); unpaired t test; error bars are s.e.m.).

Fwe is also expressed at higher levels in gustatory receptor neurons and leg taste bristle chemosensory neurons than in many other cell types (Supplementary Fig. 4). These sensory neurons are located in taste sensilla of the *D. melanogaster* labellum and legs, which are open to the external environment and allow flies to taste chemical compounds^51^. The labellum is a major taste organ in *D. melanogaster* and contains a total of 31 sensilla (Extended Data Fig. 8a). Most sensilla contain both a neuron that detects sweet compounds and a neuron that detects bitter compounds. Neuronal activity can be examined through electrophysiological recording of individual sensilla (Fig. 4c). We exposed *D. melanogaster* to a mixture of sucrose and SAIP for 7 days and then recorded the neuronal activity of each labellum sensilla to a solution of sucrose (a sweet compound) or lobeline (a bitter compound). Exposure to SAIP reduced responsiveness to the sucrose solution in most sensilla (Fig. 4d). Responses to lobeline appear to be less affected (Extended Data Fig. 8b). To confirm this finding, we further recorded neuronal activity of flies that survived to day 12. We found that most sensilla lost responses to sucrose at this point, yet many of them still maintained their responses to lobeline (Extended Data Fig. 8c). These findings revealed that SAIP can reduce the ability of gustatory neurons to sense external compounds before death. The fact that not all taste neurons are affected to the same degree suggests that the effect is specific, although the impact on fly behaviour remains to be investigated.

Besides neurons, Fwe is also expressed in haemocytes, the primary immune cells in *D. melanogaster* (Supplementary Fig. 4). We isolated haemocytes that express GFP from a transgenic *D. melanogaster* line (Hml-gal4; UAS-GFP) and incubated these haemocytes with SAIP for 24 h, which led to shrinkage and eventual death of cells (Fig. 4e). When Fwe expression was knocked down by RNA interference (RNAi)^41^, these haemocytes became resistant to SAIP (Fig. 4e).

To further examine whether SAIP can suppress immune responses in vivo, we used non-pathogenic *E. coli* as a surrogate, which normally can be cleared by the immune system in *D. melanogaster* (Fig. 4f). Neither injection of 1 fmol SAIP nor injection of *E. coli* alone caused death of *D. melanogaster* (Fig. 4f), but injection of SAIP 24 h before injection of *E. coli* resulted in death of ~50% of *D. melanogaster* (Fig. 4f). These findings suggest that SAIP, at sublethal levels, can suppress immune responses against invading bacteria in *D. melanogaster*.

### SAIP^+^*Streptomyces* spp. can kill insects and digest carcasses

**Extended Data Fig. 9:**
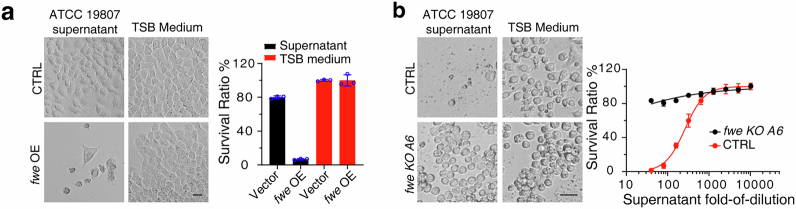
A toxigenic strain of *Streptomyces* secretes SAIP into the culture medium. **(a)** *S. hiroshimensis* strain ATCC 19807 cultured in TSB medium for one week in 29 °C. HeLa cells stably expressed *Drosophila fwe* or vector were treated with the *S. hiroshimensis* culture medium for 4 days. Cell variabilities were quantified through MTT assays. **(b)** *fwe KO* clone A6 and a control PT5/Cas9 cell expressing a scramble gRNA were subjected to ATCC 19807 culturing medium with a serial dilution for 4 days. Cell variabilities were quantified through MTT assays. Representative pictures from ATCC 19807 culture-medium-treated groups are shown on the right. Data are shown as mean ± s.d., n = 3, scale bar = 25 μm.

We next evaluated the ability of toxigenic *Streptomyces* to cause morbidity and mortality to insects. We found that the culture supernatant of a SAIP^+^ strain (*Streptomyces hiroshimensis*, ATCC 19807, which produces SAIP1.4) induced death of S2 cells and HeLa cells that express *D. melanogaster* Fwe, whereas *fwe* KO S2R+ cells and the control HeLa cells were resistant (Extended Data Fig. 9a,b). As controls, we utilized two *Streptomyces* strains related to *S. hiroshimensis* ATCC 19807 (>99% 16S rRNA identity) but not containing SAIP. We injected spores of these three strains into the haemocoel of adult *D. melanogaster*. Injection of spores of *S. hiroshimensis* ATCC 19807 resulted in the death of all flies (Fig. 4g). Remarkably, a dose as low as ~1,250 spores killed most flies within 55 h (Fig. 4g). By contrast, two control strains showed no effect on fly survival, even with 6 × 10^4^ spores (Fig. 4g). These findings demonstrate that SAIP^+^ *Streptomyces* can be highly lethal to *D. melanogaster*.

**Extended Data Fig. 10:**
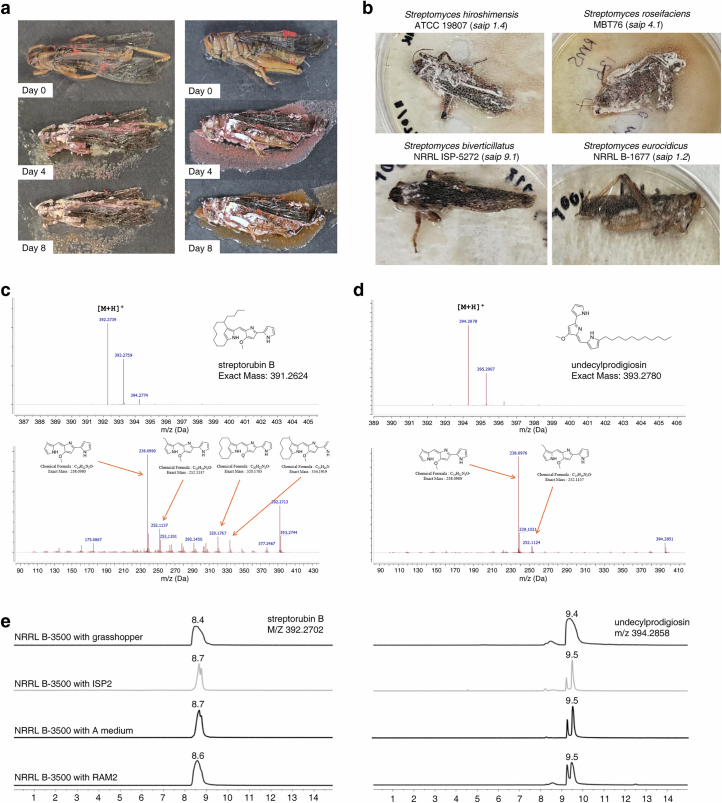
Mass spectrometry analysis of secondary metabolites produced by SAIP^+^*S. Baldacci*. **(a)** Representative images showing the robust growth of SAIP^+^ *S. baldaccii* NRRL B-3500 on the cuticle of whole grasshopper carcasses. Spores were inoculated directly on the exoskeleton of the carcass and then incubated at 28 °C for 8 days. Images show growth at days 4 and 8 following spore inoculation on day 0, for two separate carcasses. **(b)** Representative images showing four other SAIP^+^ strains (*Streptomyces hiroshimensis* ATCC 19807* (*saip*1.4) RMS0126, *Streptomyces roseifaciens* MBT76 (*saip*4.1), *Streptomyces biverticillatus* NRRL ISP-5272 (*saip*9.1), *Streptomyces eurocidicus* NRRL B-1677 (*saip*1.2) all can grow on and digest the cuticle of grasshopper carcasses similar to *S. baldaccii* NRRL B-3500. **(c-d)** High-Resolution Electrospray Ionization Mass Spectrometry (HR-ESI-MS) and MS/MS data on *S. baldaccii* NRRL B-3500 confirming the production of streptorubin B (**c**) and undecylprodigiosin **(d)**. **(e)** Extracted ion chromatograms detection of streptorubin B (m/z 392.2702) and undecylprodigiosin (m/z 394.2858) produced by *S. baldaccii* NRRL B-3500 from growth on grasshopper carcasses, ISP2, A medium, and RAM2.

We then evaluated whether toxigenic *Streptomyces* may utilize the insect carcasses as a nutrient source. Although most *Streptomyces* strains contain genes encoding chitinases that can degrade insect exoskeletons, these enzymes are thought to be used primarily for degrading fungal cell walls as part of their saprophytic lifestyle. However, recent work has reported that *Streptomyces venezuelae* grows well on insects and even on insect exoskeletons as the sole source of carbon and nitrogen^52^. All SAIP^+^ strains encode multiple chitinase genes (Supplementary Fig. 5). We thus conducted growth assays to explore the ability of a SAIP^+^ strain (*Streptomyces baldaccii* NRRL B-3500) to digest insect carcasses using dead grasshoppers as a model. This strain exhibited rigorous growth on autoclaved whole grasshopper carcasses, rapidly colonizing the exoskeleton (Fig. 4h) and, within 1 week, digested the entire insect (Extended Data Fig. 10a and Supplementary Videos 3 and 4). We further tested four more SAIP^+^ strains and all of them colonized and consumed grasshopper carcasses (Extended Data Fig. 10b).

Intriguingly, during our grasshopper growth assay, we observed an accumulation of a red-pigmented compound (Fig. 4h), suggesting the production of secondary metabolites. We conducted mass spectrometry on extracts from the insect carcass and identified two known compounds, undecylprodigiosin and streptorubin B (Fig. 4i). We confirmed both chemical structures and their production by *S. baldaccii* NRRL B-3500 through in vitro pure culture broth fermentation followed by mass spectrometry (Extended Data Fig. 10c–e). These prodiginine-class compounds are known to be red pigments and have antimicrobial activity^53^. Analyses of the *S. baldaccii* genome confirmed the presence of the *red* biosynthetic gene cluster (BGC) associated with prodiginine-class compounds (Supplementary Fig. 6). Interestingly, we found that this *red* BGC is only in 251 out of 67,561 actinomycete genomes that we have analysed (0.4%), yet it is present in 26 out of 30 SAIP^+^ strains (Fig. 1a and Supplementary Fig. 6).

## Discussion

Here we report the discovery of a family of insecticidal protein toxins in *Streptomyces*, with structural and functional homology to DT. The antagonistic interactions between *Streptomyces* and insects are not well characterized. Some *Streptomyces* produce metabolites that are toxic to *D. melanogaster* larvae^14^, but these metabolites possess broad DNA-damaging activity rather than target insects specifically. Avermectins, a group of macrocyclic lactone compounds derived from *Streptomyces avermitilis*, possess insecticidal activity but are not specific for insects either^54^. By contrast, the specificity of SAIP towards insects is unambiguously established by our discovery of its receptor Fwe, and this specificity of SAIP strongly suggests that toxigenic *Streptomyces* strains could be cryptic insect pathogens.

Producing exotoxins is energetically costly, and these proteins play no role in bacterial metabolism. Thus, they are predicted to serve an important role for the bacterium if maintained in the genome over large evolutionary timescales, as with SAIP within this lineage of *Streptomyces*. Some *Streptomyces* evolving a protein toxin that inhibits protein synthesis through ADP ribosylation of diphthamide residue in eEF2 probably facilitated the ability of these bacteria to kill and use insects as food, contributing to them becoming important microbial components of terrestrial ecosystems. Nevertheless, in our studies, functional characterization of SAIP was carried out using *Drosophila* as a model in the laboratory. It remains unknown what could be the routes of toxin entry and the specific infection caused by toxigenic *Streptomyces* in nature. Future ecological investigations into the natural niches of these toxigenic *Streptomyces* species will help inform on their pathogenesis processes in insects.

Many entomopathogenic bacteria possess well-characterized insecticidal proteins, such as crystalline (Cry) proteins in *Bacillus thuringiensis* (Bt)^55^, and Toxin complex (Tc) proteins and PirAB binary toxins in *Photorhabdus* and *Xenorhabdus* species^39,56,57^. Some of these insecticidal proteins are critically important for pest control, such as *Bt*Cry proteins utilized in transgenic crops. Given its distinct mode of action from Cry proteins, SAIPs have the potential to be developed as insecticidal proteins to address the emergence of resistance in pest populations^58^. These previously known insecticidal proteins are often structurally and functionally distinct from classic toxins causing human diseases. SAIP, on the other hand, represents an intriguing case where an insecticidal toxin is a close homologue of a deadly toxin to humans and shares the same mode of action. The *saip* gene has been vertically inherited within a monophyletic clade of *Streptomyces* through millions of years and is under strong stabilizing selection. Whether it could have served as the ancestral source for the eventual emergence of a deadly human toxin is an intriguing possibility, although it remains speculative.

As a rich and deep source of drugs, *Streptomyces* has been subjected to unparalleled microbiological sampling, including some pharmaceutical discovery efforts involving screening of millions of soil isolates^59^. Besides numerous small-molecule secondary metabolites, the discovery of SAIP illustrates that *Streptomyces* may have evolved many protein-based highly specialized weapons for combating other organisms. Interestingly, a family of large protein particles in *Streptomyces*, named umbrella toxin, was recently discovered and shown to target and suppress competing bacteria^60^. *Streptomyces* and insects have co-existed for over 400 Myr. The discovery of SAIP showcases that there is much to learn about the role of *Streptomyces* and their interactions with insects in nature, considering that both *Streptomyces* and insects are major components of terrestrial ecosystems and could be critical for Earth’s ecological systems.

## Methods

### Cell lines, insect strains and bacterial strains

*D. melanogaster* S2R+ cells were provided by the *Drosophila* RNAi Screening Center. A subline expressing SpCas9 and containing an attP integration site, S2R+/NPT005/MT-Cas9 (PT5/Cas9), was described previously and is available at the *Drosophila* Genomics Resource Center. *A. aegypti* Aag2 (RRID:CVCL_Z617) (referred to as ‘Aag2’), *A. coluzzii* Sua5B (RRID:CVCL_RQ24) and a derived Cas9-expressing clonal cell line (referred to as ‘Sua5B/Cas9’ in the manuscript) Sua5B-IE8-Act::Cas9-2A-Neo (CVCL_B3N3, DGRC stock number 334)^49^ were provided by the N. Perrimon laboratory. All cells were grown in Schneider’s Media (21720-024, Thermo Fisher) containing 10% fetal bovine serum (16140-071, Thermo Fisher) and 1× Penn/Strep (15070063, Thermo Fisher). To maintain Cas9 transgene, PT5/Cas9 cells were grown in 200 ng ml^−1^ hygromycin (40051, Calbiochem) and Sua5B/Cas9 cells were grown in 500 μg ml^−1^ geneticin (11811031, Thermo Fisher). HeLa (CCL-2), U2OS (HTB-96), J774A.1 (TIB-67), RAW246.7 (TIB-71), CT26 (CRL-2638) and HEK293T (CRL-3216) cells were obtained from ATCC. v39596 was obtained from the Vienna *Drosophila* Resource Center. Hml-gal4, UAS-GFP and w1118 strain flies were from the N. Perrimon laboratory.

*S. hiroshimensis* strain ATCC 19807 was obtained from the American Type Culture Collection. All other *Streptomyces* strains used in this study were obtained from the US Department of Agriculture ARS Culture Collection (NRRL). All *Streptomyces* strains were maintained as cryopreserved spore stocks in 35% glycerol at −80 °C. We routinely cultured *Streptomyces* on International *Streptomyces* Project-2 (ISP2; 0.4% (w/v) yeast extract, 1% (w/v) malt extract, 0.4% (w/v) dextrose, 2% (w/v) agar), International *Streptomyces* Project-3 (ISP3; 2% (w/v) boiled and strained white oats, 1.8% (w/v) agar (Bacto), 1 × 10^−4^% (w/v) iron (II) sulfate, 1 × 10^−4^% (w/v) manganese chloride, 1 × 10^−4^% (w/v) zinc sulfate) or soya flour mannitol (SFM; 2% (w/v) mannitol, 2% (w/v) soya flour, 2% (w/v) agar (Bacto)) medium. For liquid cultures of *Streptomyces*, we used tryptone soy broth (TSB; 1.7% (w/v) pancreatic digest of casein, 0.3% (w/v) papaic digest of soybean, 0.25% (w/v) dextrose, 0.5% (w/v) sodium chloride, 0.25% (w/v) dipotassium phosphate). All *E. coli* strains were maintained on lysogeny broth (LB; 1% (w/v) tryptone, 0.5% (w/v) yeast extract, 1% (w/v) sodium chloride) or LB plates solidified with 1.5% (w/v) agar (Bacto).

### Plasmid construction

To construct pCMV-DT/DT orthologue-C domains, fragments with stop codon from AAV70486.1 (DT-C, 1–186aa), WP_030364034.1 (SAIP2.1-C, 1–221aa), PSJ28985.1 (SAIP3.1-C, 1–229aa), WP_120757473.1 (SAIP1.1-C, 29–211aa), WP_162873017.1 (ACT-C, 1–249aa), PAU47471.1 (AT-C, 1–344aa) and WP_093864399.1 (Tli-C, 1–248aa) were amplified and cloned into the vector pEGFP-N1 between the NheI and HindIII sites through Gibson assembly (E2621, NEB).

*Drosophila fweA* (NP_648804.1), *Drosophila fweB* (NP_730072.1), human *fwe1* (NP_060056.1), human *fwe2* (NP_001129247.1), human *fwe3* (NP_001229298.1), human *fwe4* (NP_001229299.1) (human gene symbol *CACFD1*), mouse *fwe1* (NP_084138.1), mouse *fwe2* (NP_001230168.1), mouse *fwe3* (NP_001230169.1) (mouse gene symbol *Cacfd1*), *A. aegypti fwe* (aa*fwe*, XP_001657464.1), *Anopheles gambiae fwe* (ag*fwe*, XP_309964.4), *Spodoptera frugiperda fwe1* (sf*fwe1*, KAG8114371.1), *Spodoptera frugiperda fwe2* (sf*fwe2*, XP_035446521.1), red flour beetle *fwe* (tc*fwe*, XP_008193020.1), *O. abietinus fwe* (oa*fwe*, XP_012275392.1), *C. elegans* (ce*fw*e, NP_510484.1), *Manduca sexta fwe* (ms*fwe*, XP_030027411.1), *Atta colombica fwe* (ac*fwe*, XP_018044113.1) and *Zootermopsis nevadensis fwe* (zn*fwe*, XP_021914169.1) were synthesized by Twist Bio. Their sequences were cloned into pLenti CMV/TO Hygro (w214-1) (17484, Addgene) between the sites BamHI and XbaI for establishing stable cell lines. Most of these *fwe* orthologues were also subcloned into the pActin-mcherry-vhh05 vector for S2 cell expression.

### Protein purification

The full-length sequences of SAIP variants were subcloned into the sites NdeI and XhoI in the vector pET28a for expression in *E. coli* BL21(DE3). Cells were cultured in autoinduction medium at 37 °C until the optical density at 600 nm (OD_600_) reached 0.8, and the cultures were shifted to 18 °C and collected after 18 h. Cell pellets were lysed by sonication and purified as His6-tagged proteins through Ni-NTA beads (25216, Thermo Scientific).

For SAIP1.1-C large-scale protein purification, *E. coli* BL21(DE3) competent cells were transformed with pET28a vector encoding N-terminally His6-tagged SAIP1.1-C. The colonies were grown in TB medium supplemented with 0.5% glycerol, 50 μg ml^−1^ kanamycin and a few drops of the anti-foaming agent in the LEX bioreactor system (Epiphyte Three) at 37 °C. When OD_600_ reached 0.9, the cultures were induced with 0.5 mM isopropylthiogalactoside and the temperature was reduced to 18 °C. The cells were collected after ~18 h aftert induction cultivation. Cell pellets were resuspended in lysis buffer (100 mM Tris-HCl, pH 8.0, 500 mM NaCl, 10 mM imidazole, 1 mM TCEP buffer). DNAse I, lysozyme and protease inhibitor mix (EDTA-free, EASYpack, Roche) were added to the cell suspension, and the cells were lysed by sonication. The lysed sample was then ultracentrifuged at 204,471*g* for 45 min at 4 °C to remove cell debris. A pre-packed 5 ml HisTrap FF Ni-NTA column (Cytiva) was loaded with the clarified lysate. The protein was eluted at 10–125 mM imidazole. Fractions containing the protein were collected and loaded onto a Superdex200 16/60 column (Cytiva) pre-equilibrated with 20 mM Tris-HCl, pH 7.5, 300 mM NaCl and 0.5 mM TCEP buffer. After the gel filtration, fractions containing the protein were collected and concentrated to 60 mg ml^−1^ with Vivaspin filters (10 kDa MWCO, Sartorius). Protein concentration was determined by Nanodrop measurement, using a theoretical extinction coefficient of 16,960 M^−1^ cm^−1^ and absorbance 0.1% (1 g l^−1^) of 0.763, calculated with the Expasy ProtParam server.

### Crystallization

SAIP1.1-C protein (final concentration of 20 mg ml^−1^) was crystallized via sitting drop vapour diffusion with 1.2 M tri-sodium citrate, 0.1 M sodium cacodylate, pH 5.5 and 200 mM NDSB-211 (3-[(2-hydroxyethyl)(dimethyl)ammonio]propane-1-sulfonate, CAS: 38880-58-9) at 20 °C. Swiss 3-drop plates were used to set up 200 nl drops. The best diffracting crystals grew in drops with 1:3 protein:condition ratio. The crystals grew after 2–3 weeks, forming thin rectangular plates (~80 × 80 × 5 µm), and were fished and flash frozen in liquid nitrogen without added cryoprotectant.

### Data collection, structure determination and refinement

X-ray diffraction data were collected at the i24 beamline at Diamond Light Source (Didcot). The data were collected on a single crystal at 2.00 Å resolution, and were processed and scaled with XDS^61^. Batches 2,500–3,600 were excluded from the dataset due to poor quality, and the resolution was limited to 3.0 Å before further processing using AIMLESS in the CCP4 suite^62^. After careful consideration of merging statistics and comparing several alternative space groups, we chose to process the data in space group *C*222. Molecular replacement was performed with MOLREP^63^ using the AlphaFold2 (ref. ^64^) model of the SAIP1.1-C structure as the search model. Several cycles of model building and refinement were performed using Coot^65^, Refmac5 (ref. ^66^) and phenix.refine^67^, during which water was added to the model. Data processing and statistics are presented in Supplementary Table 4.

### Genome-wide CRISPR–Cas9 screening with SAIP1.1 or SAIP2.1

The *Drosophila* CRISPR genome-wide knockout library has been previously described^37,38^ and is available from Addgene (134582-4). In brief, 1 × 10^8^ PT5/S2R+ cells were transfected with an equal-parts mixture of pLib6.4 containing a genome-wide library (88,627 sgRNAs targeting 13,685 *Drosophila* genes) as well as pBS130 (26290, Addgene) using Effetene (301427, Qiagen) following manufacturer instructions. After 4 days, the transfected cell library was selected with 5 μg ml^−1^ puromycin (540411, Calbiochem) for 12 additional days, subculturing every 4 days. After the stable sgRNA*-*expressing cell library was established, 8 × 10^7^ cells were plated onto four 15-cm cell culture dishes to ensure sufficient sgRNA coverage, with each sgRNA being represented ~1,000 times. These cells were exposed to SAIP for 1 week. The surviving cells were further cultured in toxin-free medium to ultra-confluence and then subjected to subsequent rounds of screening with higher concentrations of toxins. Four rounds of screenings were performed with SAIP1.1 (2, 5,10 and 20 nM) or SAIP2.1 (20, 100, 260 and 400 pM). Aliquots of cell libraries following each selection round were collected and their genomic DNA was extracted using the Zymo Quick-gDNA Miniprep kit (D3025, Zymo Research). DNA fragments containing the sgRNA sequences were amplified by PCR using at least 1,000 genomes per sgRNA as template for each sample. PCR fragments were in-line barcoded using a previously described approach. Next-generation sequencing (Illumina NextSeq) was performed at the Biopolymers Facility at Harvard Medical School.

### Generating mutant cell lines

CRISPR knockouts in *D. melanogaster* PT5/Cas9 or *A. coluzzii* Sua5B-IE8-Act::Cas9-2A-Neo cells were made by transfecting cells with pLib6.4 (133783, Addgene) or pLib6.4B-Agam_695 (176668, Addgene) containing the following sgRNAs: Dmel_fwe1: 5’-CAGCCGTGGTATCTCAAATA-3’; Dmel_fwe2: 5’- CACGCTCAGCGTATCCTGCC-3’; Dmel_fwe3: 5’-CCGCCCATTATTCTGTGCTT-3’; Dmel_dph71: 5’-CTTGGGATGCAGATTGGCCA; Dmel_dph72: 5’- AGCTCCTCCCGGATGACAGC-3’; Acol_fwe1: 5’- ACCACCCTGATGGCCCGCCC-3’; Acol_fwe2: 5’- GTGCCGTGGCATCTGAAGTA-3’; Acol_fwe3: 5’- GTTGTACAGCCCGAACAGGA-3’; Acol_dph71: 5’-TAGATCAAAAATGGAACCCA-3’; Acol_dph72: 5’-TCAGCTCGAACCACAGACAA-3’; Acol_dph73: 5’-GGTGCTGGACCGGACGGTGG-3’, along with equal parts pBS130 using Effectene and selected in 5 μg ml^−1^ Puromycin. Single KO clones were picked for *Drosophila fwe* KO. For *Drosophila dph7* KO, two gRNAs were mixed together for transfection.

### MTT assays

The survival ratio of cells was analysed using the standard MTT (3-(4,5-dimethylthiazol-2-yl)-2,5-diphenyltetrazolium bromide) assay. In brief, cells were seeded in 96-well microplates with 1/20 initial confluence and incubated overnight. The medium was replaced with a toxin-containing medium, with threefold (HeLa) or 2-fold serial dilution (insect cell lines). The cells were exposed to SAIP for 4 days, then 8 μl MTT (5 mg ml^−1^ in PBS) was added to each well. After 3 h incubation, 100 μl 10% SDS solution was added to each well and the wells incubated overnight. The absorbance at 580 nm was recorded by a microplate reader (NEO2ALPHA, BioTek). A vehicle control without toxins was analysed in parallel. The cytotoxicity curves were analysed and fitted using Graphpad Prism 8. Phase-contrast images of cells were recorded (Olympus Ix51, ×20 objectives).

### Cell-based toxin binding assay

His6-HA-SAIP2.1 double-tagged protein was purified in *E. coli* BL21 for toxin binding assays. Cells were transfected with pEGFP-N1-*Drosophila fweA*, *Drosophila fweB*, human *fwe1*, human *fwe2* or GFP through PolyJet (SL100688, SignaGen Laboratories). After 24 h, transfected cells were seeded onto glass-bottom 96-well plates with ~60% confluence and further incubated overnight. The cell medium was replaced with His6-HA-SAIP2.1 (50 nM)-containing medium, the cells were incubated at 37 °C for 5 h, permeabilized with 0.3% Triton X-100 for 30 min, fixed with 4% paraformaldehyde for 20 min at room temperature, blocked with 10% goat serum for 1 h, then incubated with anti-HA antibodies (1:100, 2367S, Cell Signaling Technology) at 4 °C overnight, followed by incubation with anti-mouse Alexa-568-labelled secondary antibodies (1:500, A-11004, Invitrogen) for 1 h at room temperature. Fluorescence images were captured with a confocal system (Zeiss LSM880, ×20 or ×63 oil objective) and analysed in ImageJ bundled with 64-bit Java 1.8.0_172.

### Western blot

HEK293T cells were transfected with pCMV-SAIP-C or DT-C, collected and washed three times with PBS after 24 h after transfection. The cell pellets were lysed with modified RIPA buffer (50 mM Tris, pH 7.5, 1% NP40, 0.5% Triton X-100, 150 mM NaCl, 0.5% sodium deoxycholate, protease inhibitor cocktail). Cell lysates were centrifuged and the supernatants were loaded with native sampling buffer (BN2003, Thermo Fisher) or SDS sampling buffer, then subjected to SDS–PAGE or native gel runs and transferred onto a nitrocellulose membrane. The membrane was blocked with a TBST buffer (10 mM Tris, pH 7.4, 150 mM NaCl, 0.1% Tween-20) with 5% milk at room temperature for 1 h. Then, the membrane was incubated with anti-eEF2 primary antibodies (1:1,000, C-9, sc-166415, Santa Cruz) overnight at 4 °C, washed and incubated with anti-mouse HRP secondary antibodies (1:5,000, 31430, Invitrogen) for 1 h. Signals were detected using a Fuji LAS3000 imaging system.

### Adult flies micro-injection

Flies (10-day-old, male:female = 1:1) were kept on a CO_2_ pad. After anaesthetization, flies were injected intrathoracically with 100 nl of toxin diluted in PBS, *E. coli* bacteria, bacterial spores or PBS solution using Drummond capillary tubes pulled into fine tips and a Nanoject III Programmable Nanoliter Injector (3-000-207, Drummond). Injected flies were transferred to new fly food tubes and kept in a 23 °C fly incubator.

### Genomic DNA extraction and sequencing

We cultured *Streptomyces* strains in 3 ml TSB overnight and centrifuged cultures at 21,130*g* for 5 min. We then used the MasterPure Yeast DNA Purification kit (Lucigen) with addition of 1 µl Ready-Lyse Lysozyme Solution (Lucigen) and 1 µl 5 mg ml^−1^ RNase A (Lucigen) to lyse the pelleted *Streptomyces* cultures and extract genomic DNA. Libraries were prepared with a single library preparation method based on the Illumina Nextera kit and sequenced using the 2 × 150 bp paired-end Illumina NextSeq 2000 platform at the Microbial Genome Sequencing Center (Pittsburgh, PA).

### Genome assembly and core-genome phylogeny

We processed raw genomic reads using fastp 0.20.0 and assembled draft genome sequences using SPAdes 3.11.1, both using default parameters. We constructed a core-genome phylogeny using 93 full-length TIGRFAM amino acid sequences in the ‘core bacterial protein’ set (GenProp0799). We used the Reltime-branch Lengths method in MEGA-X^4^ to estimate divergence times of branches based on the split between *Streptomyces* and *Kitasatospora* ~382 Ma (CI 250–514 Ma) and the split between the two major clades of *Streptomyces* (132 Ma, CI 82–177 Ma). We calculated the GC percentage for the overall genome sequences and toxin sequences using a custom Python script. To infer ancestral state at each node in the phylogeny for *saip* presence or absence, we used Mesquite with default parameters (https://www.mesquiteproject.org)^68^. We visualized phylogenies using the Interactive Tree of Life platform^69^.

### DNA *Z*-curve analysis

To detect local changes in the composition of DNA surrounding the *saip* locus, we used *Z*-curve analysis^70^ and a segmentation algorithm, based on a quadratic divergence measure that calculates the maximum distribution in probability distributions of base composition between two segments of DNA, which halts when a defined threshold is not exceeded^71^. On the basis of tests to detect the integrated corynephage in *C. diphtheriae* C7β, we used halting parameters *t*_0_ = 100 and a minimum segment length of 1 kb.

### Identification of BGCs

We used multismash to run antiSMASH (7.0)^72^ for identification of BGCs, and BiG-SCAPE to group BGCs into gene cluster families (GCFs) using the following parameters: –mix–no_classify–include_singletons–clans-off–cutoffs 0.5. We then extracted GCFs using a custom Python script to generate presence–absence heat maps for each GCF family. We identified the *red* BGC in *Streptomyces* using Gator-GC^73^ based on a targeted search for clusters containing the following genes from the undecylprodigiosin BGC from *S. coelicolor* A3(2) (https://mibig.secondarymetabolites.org/repository/BGC0001063.5) as required proteins: *redX* (SCO5878), *redW* (SCO5879), *redV* (SCO5882), *redR* (SCO5886), *redQ* (SCO5887), *redP* (SCO5888), *redO* (SCO5889), *redN* (SCO5890), *redM* (SCO5891), *redL* (SCO5892), *redK* (SCO5893), *redJ* (SCO5894), *redI* (SCO5895) and *redH* (SCO5896). We detected this BGC in a total of 251 out of 67,561 genomes searched (0.4%), including 49,277 Actinomycetota genomes from NCBI, 1,999 bacterial MIBiG V3 entries, and 16,285 genomes from the NPDC (https://www.biorxiv.org/content/10.1101/2023.12.14.571759v2).

### *saip* genetic context and *saip* selection analysis

We extracted up to 10 kb surrounding the *saip* locus and used clinker to visualize gene synteny.

We identified protein-coding genes in all other *Streptomyces* genomes that encode *saip* using Bakta with default parameters. We identified genes encoding core bacterial proteins with hmmscan from HMMER 3.1b2 (http://hmmer.org/) using the best hits, generated alignments for each of the 93 core bacterial protein sequences using MAFFT, and converted the amino acid alignments into codon alignments with PAL2NAL. We calculated the *d*_N_/*d*_S_ ratio for each pairwise combination of corresponding sequences within the codon alignments for each group using dnds.py (https://github.com/adelq/dnds), discarding combinations where the ratio was undefined (*pS* = 0).

### Identification of CAZymes

We used hmmscan to detect CAZyme-encoding genes from the Bakta output, as above. We then counted the number of hits corresponding to cellulose, chitin, lignin and pectin active CAZymes^74^.

### Comparison of SAIP protein sequences and subtyping

A multiple sequence alignment (MSA) of the DT-like protein sequences was generated using Clustal-Omega^75^. To obtain conserved alignment blocks for phylogenetic analysis, the MSA was trimmed to remove a poorly aligned, variable 30-aa N-terminal segment and a C-terminal segment (position 475–549). The resulting trimmed alignment was then used to construct a maximum-likelihood phylogenetic tree using PhyML^76^ with the LG model of evolution and aLRT (SH-like) bootstrap statistics. Using the APE^77^ and ggtree packages (10.1111/2041-210X.12628), the original alignment and tree were imported into R and visualized with the alignment beginning at the first non-gapped position. A heat map of pairwise sequence identities was mapped onto the tree and plotted using the gheatmap function. In addition, a visualization of the protein alignment was generated using AliView^78^, with consensus amino acids shown in grey and amino acid substitutions indicated by different colours (default colour scheme). Guided by the tree structure, pairwise identities and variant profiles, we defined *n* = 8 phylogenetically distinct subgroups of DT-like toxins.

### Feeding assays and tip recording

For feeding assays, 30 µl of either SAIP1.1 (30 µM) or water was applied to the surface of an agarose food vial (1% agarose, 100 mM sucrose, 3.6% soybean flour). After the solutions were absorbed, 3-day-old *D. melanogaster* (strain w1118) male or female flies were separately transferred into the vials. Flies were moved to new vials daily, with SAIP1.1 or water added to the fresh vials each time. Lethality on the flies in each vial was monitored and recorded daily, and the data were plotted (Fig. 4b).

For tip recording assays (Fig. 4d and Extended Data Fig. 8), flies were fed with lower doses of SAIP1.1 (20 µM, 25 µl). Newly eclosed flies were cultured on standard fly food medium for 3 days. Then, 30 female and 10 male flies (strain w1118) were transferred into vials containing 1% agarose and 100 mM sucrose freshly coated with 20 µM SAIP1.1 or sterile water control. Flies were transferred into new vials with the same, freshly prepared medium every 24 h. Tip recordings were performed on mated, female flies raised on control or SAIP1.1-treated medium for 7–12 days. The reference electrode was inserted into the eye. A fine glass tastant solution was used to simultaneously deliver the taste stimulus and to record the electrophysiological activity of taste neurons. Tricholine citrate (30 mM) was used as an electrolyte. Signals were amplified (10× Syntech Universal AC/DC Probe), filtered (100–3,000 Hz with 50/60 Hz suppression), digitized with IDAC-4 (Syntech) and analysed with AutoSpike software. Responses were quantified as the number of spikes in the first 0.5 s after contact.

### Metabolite production by *S. baldaccii* NRRL B-3500

*S. baldaccii* NRRL B-3500 was cultivated separately in three distinct liquid growth medium: ISP2 (yeast extract 4 g l^−1^, malt extract 10 g l^−1^, dextrose 4 g l^−1^), A-medium (yeast extract 5 g l^−1^, peptone 5 g l^−1^, dextrose 10 g l^−1^, soluble starch 20 g l^−1^, CaCO_3_ 5 g l^−1^) and RAM2 (corn meal 4 g l^−1^, dextrose 10 g l^−1^, maltose 15 g l^−1^, cottonseed flour 7.5 g l^−1^, dry yeast 5 g l^−1^). Each culture contained 100 ml of the respective medium along with a total of 5 g of prewashed resin mix (2.5 g HP20 and 2.5 g Amberlite XAD7HP). We incubated the cultures at 30 °C with orbital shaking at 200 r.p.m. for 7 d. After incubation, we separated the resin from each culture, washed it with deionized water until clear, and subsequently eluted it in an open column using 800 ml of a 1:1 (v/v) methanol:acetone mixture. We concentrated the eluent by solvent evaporation under reduced pressure using a rotary evaporator, yielding the respective extracts. To identify and track the components of the red pigment formed on the surface of grasshoppers, we added 20 ml of HPLC-grade methanol to sterilized 50 ml conical tubes containing the sample, and left the mixture for 30 min. Subsequently, we filtered the mixture, collected the material and then dried it under reduced pressure using a rotary evaporator.

### LC–MS/MS analysis

For LC–MS/MS analysis, we reconstituted the extracts in HPLC-grade methanol to a uniform concentration of 0.5 mg ml^−1^. We filtered all extract samples through a 0.20 µm syringe filter for instrumental analysis. We analysed the prepared solutions using liquid chromatography–mass spectrometry (LC–MS). High-resolution electrospray ionization (HR-ESI) mass spectra with collision-induced dissociation (CID) MS/MS were acquired using an Agilent 6550 LC-qTOF mass spectrometer coupled with an Agilent 1290 uHPLC system. Metabolite separation was performed on an Agilent ZORBAX Eclipse Plus C18 column (100 × 2.1 mm) under the following chromatographic conditions: 98% solvent A (0.1% formic acid in water) from 0 to 0.5 min, followed by a linear gradient to 95% solvent B (0.1% formic acid in acetonitrile) over 10 min at a flow rate of 0.4 ml min^−1^. The mass spectrum was obtained in positive ion mode, and the full scan data ranged from 150 to 1,500 *m*/*z*. The LC–MS/MS data were exported as a .mgf file using Agilent MassHunter Qualitative Analysis software and uploaded to the GNPS platform (https://gnps.ucsd.edu/ProteoSAFe/static/gnps-splash.jsp) for metabolite dereplication. We compared the generated spectra against GNPS spectral libraries, with matches filtered on the basis of a minimum score threshold of 0.7 and at least 5 matched peaks. The dereplication results confirmed the presence of streptorubin B and undecylprodigiosin in all samples. Streptorubin B (*m*/z 392.2702, [M + H]^+^) was detected within the retention time range of 8.4–8.7 min through the extracted ion chromatogram, while undecylprodigiosin (*m*/*z* 394.2858, [M + H]^+^) was detected at 9.4–9.5 min.

### Growth assay on grasshoppers

To evaluate the ability of SAIP^+^ *Streptomyces* strains to digest insects as food, we conducted growth assays using dead grasshoppers as the sole carbon and nitrogen source. Dead grasshoppers (Fluker’s fresh feeder) were autoclaved for 15 min, removing any potential for other microbes to contribute to the breakdown of the insect in our assays. Five SAIP^+^ strains were available and tested: *S. baldaccii* NRRL B-3500, *Streptomyces biverticillatus* NRRL ISP-5272, *Streptomyces eurocidicus* NRRL B-1677, *S. hiroshimensis* ATCC 19807* RMS0126 and *Streptomyces roseifaciens* MBT76. Briefly, whole grasshoppers were inoculated through addition of 10 ml of a 10^6^ spores per ml solution in a sterile Petri dish. Spore stocks were generated from growth on solid ISP2 medium at 28 °C. Inoculated grasshoppers were incubated for 7 days at 28 °C. Solid-state growth assays were replicated 5 times. We also conducted additional tests in liquid phase to quantify biomass loss, with 5 ml of spore stock solution added to 5 ml of autoclaved tap water in 50 ml sterile conical tubes and incubated for 18 days at 28 °C. Grasshoppers were dried before weighing to remove water.

### Bioinformatics analysis and data visualization

All bioinformatics statistical analyses were performed in R and the packages are described in the preceding sections. We generated graphics using ggplot2 with some cleanup and assembly in InkScape.

### Sample sizes

No statistical methods were used to pre-determine sample sizes, but our sample sizes are similar to those reported in our previous publication^39^. Data distribution was assumed to be normal, but this was not formally tested. There was no randomization in the organization of the experimental conditions. Data collection and analysis were not performed blind to the conditions of the experiments. No animals or data points were excluded from our analyses.

### Reporting summary

Further information on research design is available in the Nature Portfolio Reporting Summary linked to this article.

## Supplementary information

Supplementary Information Supplementary Figs. 1–6 and Supplementary Tables 1–6.

Reporting Summary

Supplementary Video 1 SAIP (Supplementary Video 1) or sterile water control (Supplementary Video 2) was added and absorbed daily to the surface of an agarose food vial containing *D. melanogaster* (strain w1118). By day 5, *D. melanogaster* exposed to SAIP developed paralysis and reduced mobility.

Supplementary Video 2 SAIP (Supplementary Video 1) or sterile water control (Supplementary Video 2) was added and absorbed daily to the surface of an agarose food vial containing *D. melanogaster* (strain w1118). By day 5, *D. melanogaster* exposed to SAIP developed paralysis and reduced mobility.

Supplementary Video 3 *Streptomyces baldaccii* NRRL B-3500 (Supplementary Video 3) or sterile water control (Supplementary Video 4) was added to sterile grasshoppers and incubated for 7 days before vortexing to illustrate degradation of the dead insect.

Supplementary Video 4 *Streptomyces baldaccii* NRRL B-3500 (Supplementary Video 3) or sterile water control (Supplementary Video 4) was added to sterile grasshoppers and incubated for 7 days before vortexing to illustrate degradation of the dead insect.

## Source data

Source Data Fig. 1 A list of strain information.

Source Data Fig. 4 Experimental information for Fig. 4.

Source Data Extended Data Fig. 4 Uncropped blot scans for Extended Data Fig. 4.

## Data Availability

All biological materials are available upon request from the co-corresponding authors. Crystallographic data statistics are summarized in Supplementary Table 4. The atomic coordinates and structure factors have been deposited in the Protein Data Bank (PDB) under accession code 9QE9. Source data are provided on strain information (for Fig. 1a), experimental conditions (for Fig. 4) and uncropped blot scans (for Extended Data Fig. 4). All mass spectrometry raw data generated in this study are publicly available through the GNPS MassIVE repository under accession number MSV000100974. The dataset includes all raw LC–MS files used for metabolite analysis. Detailed descriptions of all raw files are provided in Supplementary Table 6. Genome sequences generated from this work are deposited under BioProject accession no. PRJNA1403066. The raw and derived datasets for all computational analyses are available in figshare at 10.6084/m9.figshare.31073734 (ref. ^79^). Source data are provided with this paper.

## Code availability

The scripts necessary to replicate this work are available in GitHub at https://github.com/reedstubbendieck/saip.
