# Author Correction: *Streptomyces* produce a diphtheria toxin-like exotoxin that targets insects

**DOI:** 10.1038/s41564-026-02379-3

**Published:** 2026-05-14

**Authors:** Ying Xu, Reed M. Stubbendieck, Raghuvir Viswanatha, Ajda Krč, Lisa S. Baik, Won Se Suh, Yanhui Hu, Huan Wang, Linxiang Yin, Enzo Mameli, Anne van der Meij, John R. Carlson, Andrew C. Doxey, Pål Stenmark, Norbert Perrimon, Cameron R. Currie, Min Dong

**Affiliations:** 1 Department of Urology, Boston Children’s Hospital, Boston, MA USA; 2 Department of Surgery and Department of Microbiology, Harvard Medical School, Boston, MA USA; 3 State Key Laboratory of Animal Biodiversity Conservation and Integrated Pest Management, Institute of Zoology, Chinese Academy of Sciences, Beijing, China; 4 Department of Microbiology and Molecular Genetics, Oklahoma State University, Stillwater, OK USA; 5 Department of Bacteriology, University of Wisconsin-Madison, Madison, WI USA; 6 Department of Genetics, Blavatnik Institute, Harvard Medical School, Boston, MA USA; 7 Howard Hughes Medical Institute, Harvard Medical School, Boston, MA USA; 8 Department of Biochemistry and Biophysics, Stockholm University, Stockholm, Sweden; 9 Department of Molecular, Cellular and Developmental Biology, Yale University, New Haven, CT USA; 10 Department of Entomology and Nematology, University of California Davis, Davis, CA USA; 11 Department of Biochemistry and Biomedical Sciences, McMaster University, Hamilton, Ontario Canada; 12 Department of Biochemistry, University of Toronto, Toronto, Ontario Canada; 13 Department of Biology, University of Waterloo, Waterloo, Ontario Canada

**Keywords:** Bacterial toxins, Pathogens

Correction to: *Nature Microbiology* 10.1038/s41564-026-02315-5, published online 06 May 2026.

In the version of this article initially published, due to a figure preparation mistake, there was an error in Fig. 2c where the image shown as *fwe* A10 cells was an inadvertent duplicate of the *fwe* A11 image directly adjacent. The figure is now amended in the HTML and PDF versions of the article.

