# Supplementary material for: Streptomyces produce a diphtheria toxin-like exotoxin that targets insects
Source: Nat Microbiol. 2026 Apr 30;11(5):1271–85. doi: 10.1038/s41564-026-02315-5 (PMC13171429; doi:10.1038/s41564-026-02315-5)
Supplement: Supplementary file 1 — Supplementary Figs. 1–6 and Supplementary Tables 1–6. [file 41564_2026_2315_MOESM1_ESM.pdf]

nature microbiology

Supplementary information

https://doi.org/10.1038/s41564-026-02315-5

Streptomyces produce a diphtheria toxin-like exotoxin that targets insects

In the format provided by the authors and unedited

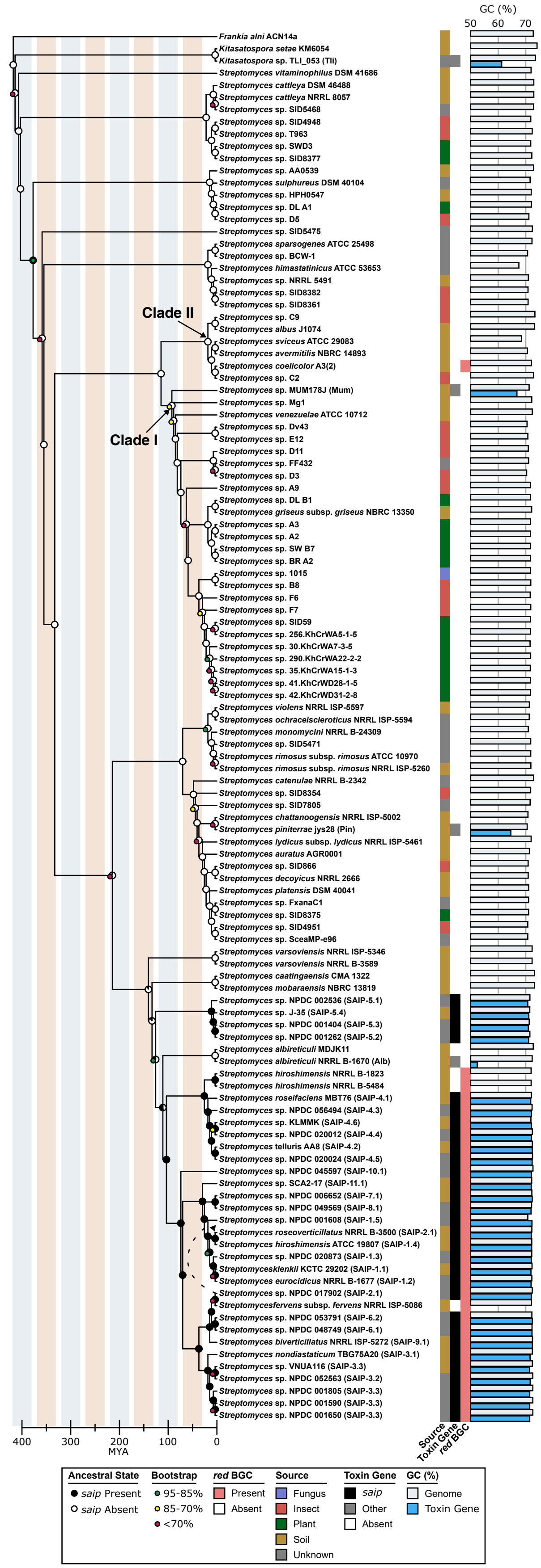

Supplementary Fig. 1. Core genome phylogeny analysis for SAIP+ strains.

Fig 1a is a subset of this tree shown here. Briefly, phylogenetic tree built from 93 conserved single copy bacterial genes (GenProp0799). The phylogeny was rooted on *Frankia alni ACN14a*. The branch lengths indicate Reltime-estimated divergence times. These divergence times were calculate based on the split between *Kitasatospora* and *Streptomyces* (382 Mya, CI: 250 to 514 Mya) and the split between the two major clades of *Streptomyces* (132 Mya, CI: 82 to 177 Mya). Ancestral state reconstruction probabilities calculated using Mesquite and bootstrap support are indicated at the nodes. A potential LGT event was determined through reconciliation with the *saip* gene tree using AnGST and the directionality of transfer is indicated with dashed and curved arrow. Bootstrap values >95% are not shown. The boxes to the right of the taxa indicate the strain source, the presence or absence of a DT homologue encoded in the genome, and the presence or absence of the red BGC. The identity of the corresponding toxin is listed in parentheses next to the strain. The grey and blue bars on the right indicate the GC content of the genome and, if applicable, the toxin-encoding gene, respectively.

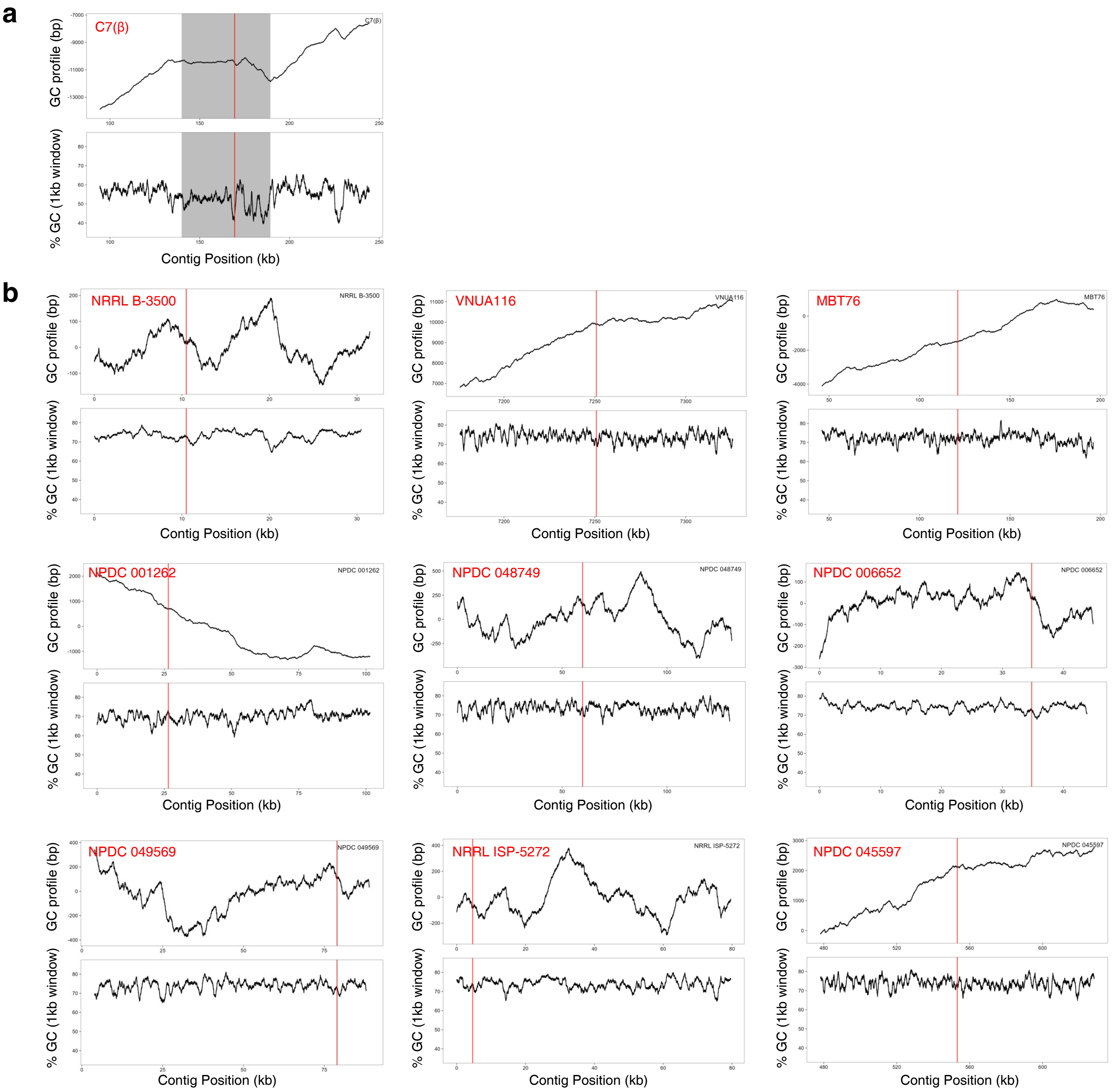

Supplementary Fig. 2. DNA Z-curve segmentation analysis around *saip* gene.

(a) GC profiles and GC content for *Corynebacterium diphtheriae* C7(β). The top panel shows the negative cumulative GC profile near the toxin-encoding gene. The bottom panel shows the GC content in 1 kb windows. The red line indicates the location of the toxin-encoding gene. Genomic segments (GC Profile halting parameter t0 = 100, minimum segment length = 1 kb) with distinct GC content are indicated by gray shading. The distinct segment corresponds to the integrated corynephage (132 to 170 kb).

(b) GC profiles and GC content for the indicated SAIP+ *Streptomyces* strains. The top panel shows the negative cumulative GC profile near the toxin-encoding gene. The bottom panel shows the GC content in 1 kb windows. The red line indicates the location of the SAIP-encoding gene. Genomic segments (GC Profile halting parameter t0 = 100, minimum segment length = 1 kb) with distinct GC content are indicated by gray shading.

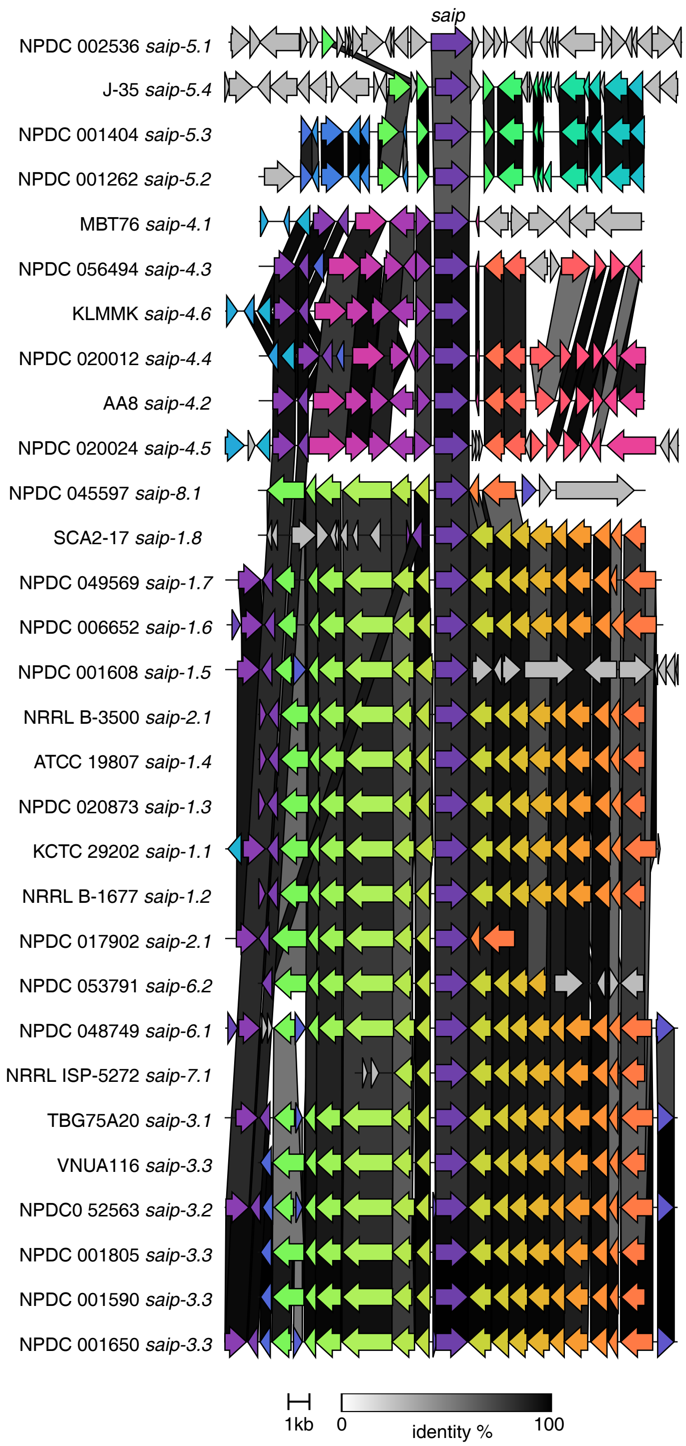

Supplementary Fig. 3. Synteny of the *saip* locus in toxigenic *Streptomyces*.

The genomic regions within 10 kb of *saip* were extracted and aligned. The scale bar indicates the size of the genomic regions. Shared colors indicate homologous genes. Connections between genes represent sequence identity, as indicated by the scale at the bottom. For NPDC 017902 and NRRL ISP-5272, *saip* was located near a contig edge.

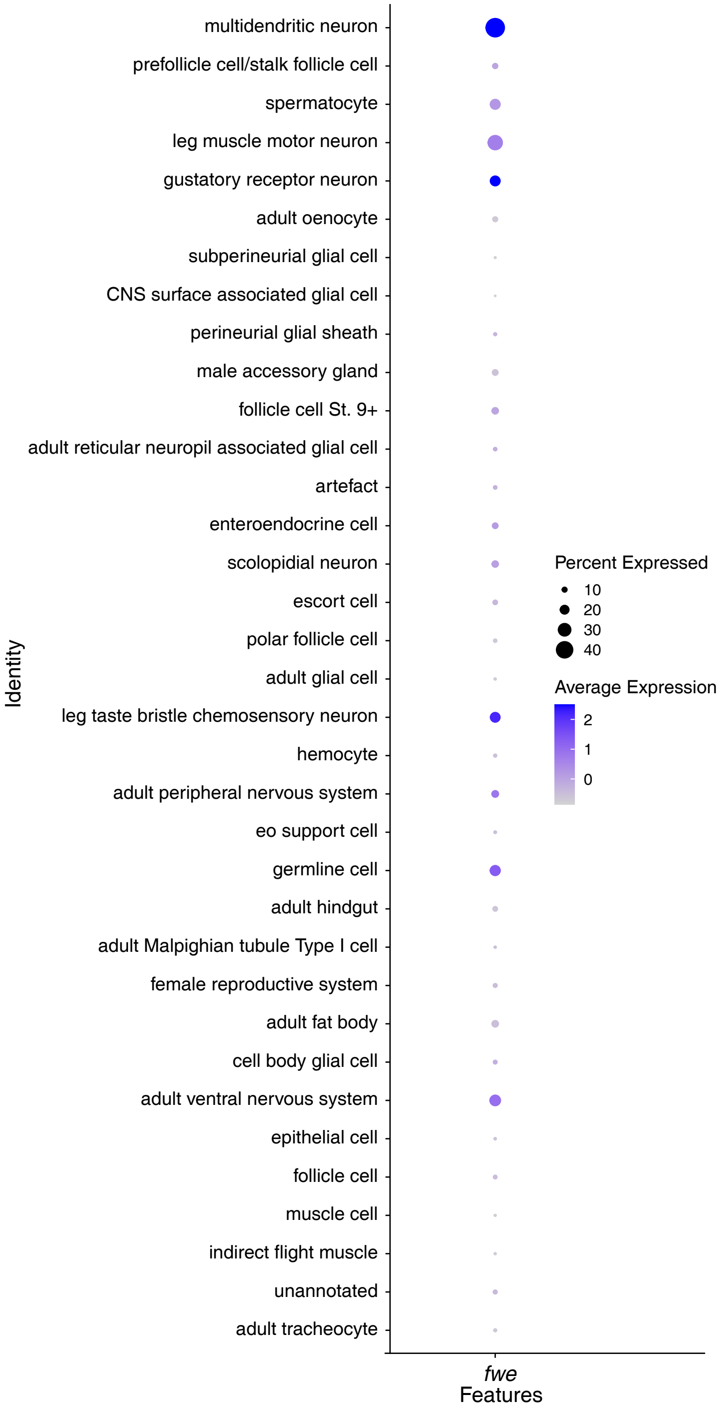

Supplementary Fig. 4. Single-cell RNA expression analysis of *fwe* across *Drosophila* body.

Fly Cell Atlas consortium generated the transcriptomic atlas of the entire adult *Drosophila* body at single cell resolution. The datasets contain over 580 K cells with two sequencing strategies: droplet-based 10x Genomics and plate-based Smart-seq261. To understand the expression pattern of *fwe*, we retrieved the 10x scRNA-seq datasets obtained from FCA (https://scope.aertslab.org/#/FlyCellAtlas/*/welcome). The scaled average expression and the percent of cells expressing fwe in each cell types were calculated and visualized using dot plot with the size for percent cells expression and the darkness for average expression.

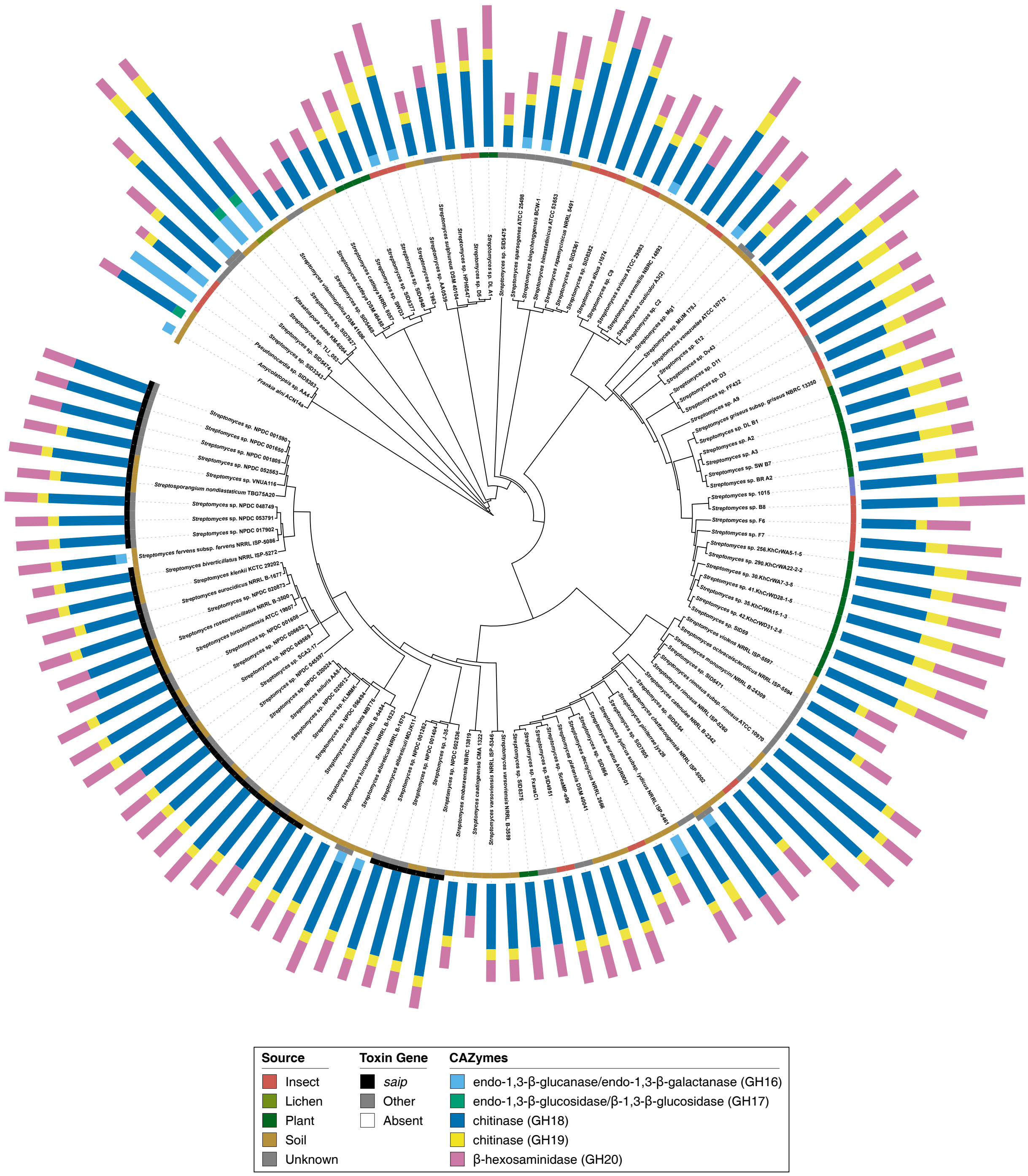

Supplementary Fig. 5. SAIP+ *Streptomyces* strains encode predicted CAZymes for chitin degradation.

Core-genome phylogeny of *Streptomyces* and closely related *Actinobacteria*. The phylogenetic tree was built from 93 conserved single copy bacterial genes (GenProp0799). The phylogeny is rooted on the lineage containing *Amycolatopsis* sp. AA4, *Frankia alni* ACN14a, and *Pseudonocardia* sp. SID8383. Branch lengths were transformed to be proportional to the root. The isolation source is indicated by the inner ring and the outer ring indicates the presence of a *saip* encoded in the genome. The stacked bar plots indicate the number of predicted CAZymes involved in chitin degradation encoded by the corresponding strain.

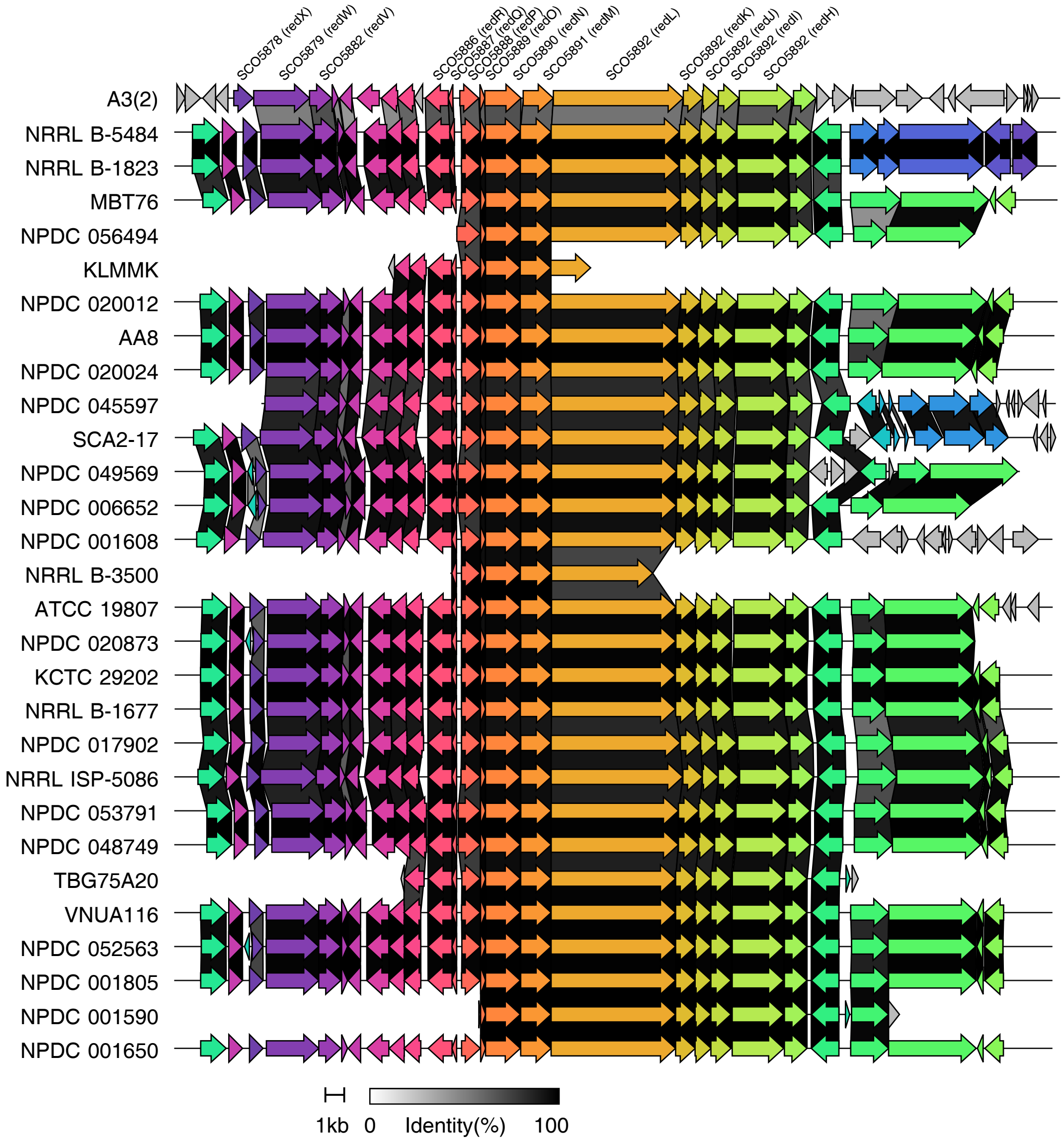

Supplementary Fig. 6. Genome distribution of the *red* BCG.

The genomic regions within 10 kb of the red BGC were extracted and aligned. The scale bar indicates the size of the genomic regions. Shared colors indicate homologous genes. The corresponding SCO accession numbers for *Streptomyces coelicolor* A3(2) are shown above the arrows. Connections between genes represent sequence identity, as indicated by the scale at the bottom. For KLMMK. NRRL B-3500, TBG75A20, and NPDC 001590, the red BGC was located near a contig edge.

Organism	Protein ID		Name	Report Sample Collection	Location
Streptomyces klenkii KCTC 29202	WP_120757473.1	*	SAIP1.1	Marine sediment	Turkey: black sea
Streptomyces roseoverticillatus NRRL B-3500	WP_030364034.1	*	SAIP2.1	Soil	Italy: Arcinazzo
Streptosporangium nondiastaticum TBG75A20	PSJ28985.1	*	SAIP3.1	Forest soil	India: Kerala
Streptomyces sp. NRRL B-1677	WP_194959766.1		SAIP1.2	Not Available	Japan: Okami (NIHJ)
Streptomyces roseifaciens MBT76	WP_107105579.1	*	SAIP4.1	Soil	China: Xi'an
Streptomyces telluris AA8	WP_256791793.1		SAIP4.2	Garden Soil	Thailand: Bangkok
Streptomyces sp. VNUA116	WP_302859207.1		SAIP3.3	Soil in a banana plantation	Vietnam
Streptomyces sp. KLMMK	WP_335581086.1		SAIP4.6	Soil	USA: Illinois
Streptomyces sp.TLI053	WP_159425252.1	*	Tli	Not Available	Not Available
Streptomyces piniterrae jys28	TJZ58907.1	*	Pin	Rhizosphere, Pinus yunnanensis	China: Kunming
Austwickia chelonae LK16-18	WP_162873017.1	*	ACT2	Crocodile lizard	China: Shaoguan
Austwickia sp. TVS 96-490-7B	WP_219106995.1	*	ACT3	Cutaneous lesion from Crocodylus porosus	Australia: Darwin
Streptomyces albireticuli NRRL B-1670	PAU47471.1	*	AT	Soil	Indonesia: Java, Jakarta
Seinonella peptonophila DSM 44666	WP_073156187.1	*	Pep	Soil	Japan: Yamanashi Prefecture
Streptomyces sp.MUM 178J	MCH0551590.1		Mum	Soil	Malaysia: Kuching
Klebsiella aerogenes 2022GN-00122	EIZ2913133.1		Aer	Not Available	USA
Streptomyces sp. NBC_00224	WP_302859207.1		Nbc	Soil	Finland: Helsinki
Streptomyces gamaensis DT85	MFC5720489.1		Gam	Not Available	Not Available

Supplementary Table 1. Summarization of the information about DT-like proteins and bacteria in NCBI.
We initially used DT as the bait, and searched for homologs using Blastp. After we got DT homologs, we then used these hits as queries to further conduct Blastp search in NCBI. All DT homolog sequences were then manually examined and selected based on the criteria that all three-domains must be present. * marks the sequences previously reported[27-29]

	DT	SAIP1.1	Tli	Pin	ACT2	ACT3	AT	Pep	Mum	Aer	Nbc	Gam
DT	100											
SAIP1.1	23.76	100										
Tli	21.85	49.79	100									
Pin	26.21	37.74	33.69	100								
ACT2	40	30.72	24.95	27.43	100							
ACT3	42.16	26.02	25.45	26.72	48.98	100						
AT	19.14	22.48	21.76	21.15	22.63	21.75	100					
Pep	22.31	24.26	22.35	26.61	25.5	23.06	31.21	100				
Mum	25	59.29	45.49	40.93	31.22	27.27	22.35	26.24	100			
Aer	26.56	29.66	27.29	29.62	28.13	29.06	22.76	22.33	30.86	100		
Nbc	25.51	49.79	41.75	38.98	30.56	26.19	23.01	24.61	62.94	29.6	100	
Gam	24.43	63.71	42.74	37.56	30.33	28.51	25.06	26.47	60.33	29.66	51.25	100

Supplementary Table 2. Global pairwise comparisons among selected DT-like proteins.
The value is % identity. Comparisons were done using EMBL-EBI Clustal Omega (https://www.ebi.ac.uk/jdispatcher/msa/clustalo?order=input).

	SAIP1.1	SAIP2.1	SAIP3.1	SAIP1.2	SAIP4.1	SAIP4.2	SAIP3.3	SAIP4.6	DT
SAIP1.1	100								
SAIP2.1	95.71	100							
SAIP3.1	96.73	97.35	100						
SAIP1.2	99.39	95.73	96.75	100					
SAIP4.1	93.27	91.87	93.50	93.29	100				
SAIP4.2	92.45	92.07	92.89	92.48	96.34	100			
SAIP3.3	96.94	97.36	99.59	96.95	93.29	92.89	100		
SAIP4.6	91.22	89.84	91.45	91.06	96.95	96.75	91.46	100	
DT	26.67	27.00	25.61	26.77	27.23	27.00	26.54	27.69	100

Supplementary Table 3. Global pairwise comparisons among selected SAIPs and DT. The value is % identity.

Supplementary Table 4. Data collection and refinement statistics (molecular replacement)

	**SAIP-C domain**
**Data collection and processing**	
Station	Diamond Light Source, i24
Wavelength (Å)	0.6702
Temperature (K)	100
Detector	Dectris Eiger X 9M
Total rotation range (°)	360
Rotation per image (°)	0.1
Exposure time per image (s)	0.005
Space group	C 222
Cell dimensions	
a, *b*, *c* (Å)	71.2, 153.5, 71.2
α, β, γ (°)	90, 90, 90
Mosaic spread (°)	0.337
Resolution (Å)	29.9 - 3.0 (3.18 - 3.0)
Total Reflections	65087 (12193)
Unique Reflections	7385 (1286)
R_{merge}	0.364 (0.782)
R_{meas}	0.411 (0.873)
R_{pim}	0.189 (0.386)
$I / \sigma I$	7.8 (3.9)
Completeness (%)	91.1 (100.0)
Multiplicity	8.8 (9.5)
CC half	0.932 (0.702)
Overall Wilson B factor (A^2)	19.80
**Refinement**	
Resolution range (Å)	29.4 - 3.0
No. reflections all/free	7327 (361)
R_{work} / R_{free}	0.2493/0.3005
No. of non-H atoms	2675
Protein residues	357
Waters	16
Average *B*-factors (Å^2)	31.0
Macromolecules	31.4
Water	11.7
R.m.s. deviations	
Bond lengths (Å)	0.001
Bond angles (°)	0.380
Ramachandran plot	
Favored regions (%)	98.3
Allowed regions (%)	1.7
Outliers (%)	0.0
Rotamer outliers (%)	0.0
Unmodelled/incomplete residues per chain	Chain A: 32<br>Chain B: 33

*Values in parentheses are for highest-resolution shell.

Untreated, top 25 sgRNAs

Rank	Sequence	Gene	Symbol	% of library
1	TGCTACAGTCTCCGAATCCA	FBgn0052262	CG32262	0.48
2	CATATCCTGTGGAGCGCCTT	FBgn0028381	Decay	0.30
3	CACGTACTCCCCAATATCAA	FBgn0011260	Sema2a	0.27
4	GAACAGGCACGTGATCAGTT	FBgn0024994	Ugalt	0.19
5	GACCGAGGGCATCTTGCTGA	FBgn0031631	CG3225	0.12
6	TTGATTTGTCGGATGCTCCT	FBgn0037794	CG6254	0.11
7	CGCATCCTGAACGGCACTTG	FBgn0262937	Rabex-5	0.09
8	TGAGTGAAGTTCTTTCGGTG	FBgn0052457	CG32457	0.09
9	TGCCCTGATCACCCTGTTGG	FBgn0034315	CG5721	0.09
10	ATGACGGATCTTATCAGGCA	FBgn0031662	CG3792	0.08
11	TCATCTCTGCACGCACCTTG	FBgn0025616	CG13359	0.08
12	TCAGTCGGGAGAGCGAGGTT	FBgn0013753	Bgb	0.08
13	CGAGACGTTCCGCATGCAGA	FBgn0031117	GstT3	0.08
14	TGCGGCCAATAAATGGATAC	FBgn0039029	CG4704	0.08
15	CAGAGCAGCCTTCGCGGTAC	FBgn0025463	Bap60	0.07
16	CGGGCCGTTCCCAAACCTGG	FBgn0033479	PIG-N	0.07
17	CGTGACCTTCAGTTCAGGCC	FBgn0034693	flap	0.07
18	TCTCACCTGCGATTCGCCAC	FBgn0029768	SPR	0.07
19	TGTCGTCCGCCAGGGACTTT	FBgn0037191	CG14448	0.06
20	TAGCTCCTTTCCCTTCAACG	FBgn0022069	Nnp-1	0.06
21	TCTGGACTGCAAGAACGCCA	FBgn0030519	CG11151	0.06
22	TGGTCGTGTCGAGGGTTAGC	FBgn0002552	lin	0.06
23	TGAACCACGAGGTTTGCCCT	FBgn0033763	CG8646	0.06
24	TGGAGCATAAGGATGTTATT	FBgn0267432	kl-3	0.05
25	TGGGTAGAAGTCGTGCGACT	FBgn0261552	ps	0.05

SAIP1.1, top 25 sgRNAs

Rank	Sequence	Gene	Symbol	% of library
1	CAGCCGTGGTATCTCAAATA	FBgn0261722	Fwe	39.27
2	TGCTCACATAGAATCCACCG	FBgn0050259	CG30259	18.64
3	GCTCAGCGTATCCTGCCTGG	FBgn0261722	Fwe	16.96
4	TCCATCCGCCGATCGTTCGA	FBgn0011606	Klp3A	3.60
5	GCCGGTGCCCAGCGGCATGA	FBgn0020930	Dgkepsilon	3.41
6	TGTGTGGGTGCAGCGGCTCA	FBgn0034061	Ufc1	1.44
7	CTATGCACCCACTTGGTCCT	FBgn0050293	Cht12	1.35
8	GACCTGGTACTCGTAACCAT	FBgn0085300	Cpr65Ay	0.92
9	TCTTGAGTACGAGTCACACA	FBgn0038826	Syp	0.74
10	TAGTTGTTGACCATTACTTC	FBgn0032906	RPA2	0.48
11	TTGTTCGGACGCACAGCTCT	FBgn0038690	CG11703	0.39
12	GTGCGGCTTGGCGGGCGTTC	FBgn0038950	CG5382	0.38
13	CTCGGACGGTAGAGTCCACT	FBgn0034824	Klp59C	0.35
14	CGGCTGTTGATCGTTGTAGG	FBgn0000477	DNaseII	0.32
15	AAAGGATTGCGTCCGGGTGG	FBgn0262881	MtkI	0.32
16	TGGGCAATCGAAGTCTGTGT	FBgn0263982	CG43731	0.31
17	CCCTGCCCGTCAACGTCAAA	FBgn0046247	CG5938	0.31
18	GGTCAGAACCGGCTGGGCGT	FBgn0004778	Ccp84Af	0.30
19	TCTGGGTTTCTTGCGTGAGG	FBgn0035555	CG13720	0.26
20	GCATGGTTCGACTTCTTAAT	FBgn0037230	Nepl11	0.26
21	CCGAATCCGCCCTTGTGGAC	FBgn0033339	Sec31	0.25
22	GGACGCGTTCATCAAGAATG	FBgn0034325	CG18539	0.24
23	ATTTGTGGTGATGCTCTTGG	FBgn0261722	Fwe	0.22
24	AATATTCCGTATCCTCGCTG	FBgn0029892	CG3184	0.21
25	CAGTTAGCCGGGCTAACAGG	FBgn0261807	CG42753	0.21

SAIP2.1, top 25 sgRNAs

Rank	Sequence	Gene	Symbol	% of library
1	CAGCCGTGGTATCTCAAATA	FBgn0261722	Fwe	17.50
2	GCTCAGCGTATCCTGCCTGG	FBgn0261722	Fwe	6.97
3	TCCATCCGCCGATCGTTCGA	FBgn0011606	Klp3A	6.91
4	TGCTCACATAGAATCCACCG	FBgn0050259	CG30259	5.72
5	AATATTCCGTATCCTCGCTG	FBgn0029892	CG3184	1.50
6	TGTGTGGGTGCAGCGGCTCA	FBgn0034061	Ufc1	1.11
7	GCCGGTGCCCAGCGGCATGA	FBgn0020930	Dgkepsilon	1.01
8	CATGCCCAGGATATCGGCGA	FBgn0024321	NK7.1	0.99
9	CTATGCACCCACTTGGTCCT	FBgn0050293	Cht12	0.92
10	GTGCGGCTTGGCGGGCGTTC	FBgn0038950	CG5382	0.70
11	CCGAATCCGCCCTTGTGGAC	FBgn0033339	Sec31	0.64
12	TCGCCGAAGGCACTGTGAGT	FBgn0036566	ClC-c	0.58
13	TACTTTCACCATAGCTGCAC	FBgn0038570	Prx5	0.50
14	CTTAGTTTGGGTCCGATGAG	FBgn0028663	VhaM9.7-b	0.41
15	TGCTCACCTCAACGGTTCCT	FBgn0037661	Ada	0.41
16	TCGGGTGTGATGGACAACTT	FBgn0052040	CG32040	0.31
17	GACCTGGTACTCGTAACCAT	FBgn0085300	Cpr65Ay	0.30
18	TGTCCGCCGTCATTCTTGTC	FBgn0031855	meng	0.26
19	TCTTGAGTACGAGTCACACA	FBgn0038826	Syp	0.24
20	ACGGGTCACCTGCTTGATCA	FBgn0015221	Fer2LCH	0.23
21	CAGCCACTTCATGTCGAGAA	FBgn0029892	CG3184	0.22
22	TCATCCGATTCGATTAGCTC	FBgn0261832	CG42764	0.22
23	GATTGTCAAACTGAGCTTAC	FBgn0041235	Gr59c	0.22
24	TCTGGGTTTCTTGCGTGAGG	FBgn0035555	CG13720	0.18
25	CTCGGACGGTAGAGTCCACT	FBgn0034824	Klp59C	0.18

Supplementary Table 5. Top-ranked genes identified in genome-wide CRISPR screens.
List of most prevalent 25 sgRNAs (out of 84,563) in cell mixes following the last round of selection with SAIP1.1, SAIP2.1, or untreated control. Independent sgRNAs targeting some top hits are highlighted by different colors.

File name	Sample type	Condition	Extraction method	Description
WSS_SAIP2_117+GH_YW_01.mzXML	Streptomyces extract	Grasshopper-associated	YW	Extract from Streptomyces grown on grasshopper
WSS_SAIP2_117+GH_NW_01.mzXML	Streptomyces extract	Grasshopper-associated	NW	Extract from Streptomyces grown on grasshopper
WSS_SAIP2_117_small_ISP2_01.mzXML	Monoculture	ISP2 medium	Resin extract	Streptomyces monoculture extract
WSS_SAIP2_117_small_ISP2_02.mzXML	Monoculture	ISP2 medium	Resin extract	Streptomyces monoculture extract
WSS_SAIP2_117_small_A_01.mzXML	Monoculture	A medium	Resin extract	Streptomyces monoculture extract
WSS_SAIP2_117_small_A_02.mzXML	Monoculture	A medium	Resin extract	Streptomyces monoculture extract
WSS_SAIP2_117_small_RAM2_01.mzXML	Monoculture	RAM2 medium	Resin extract	Streptomyces monoculture extract
WSS_SAIP2_117_small_RAM2_02.mzXML	Monoculture	RAM2 medium	Resin extract	Streptomyces monoculture extract
WSS_BLK+GH_YW_01.mzXML	Control	Grasshopper only	YW	Grasshopper extract without bacteria
WSS_BLK+GH_NW_01.mzXML	Control	Grasshopper only	NW	Grasshopper extract without bacteria
WSS_BLK_ISP2_01.mzXML	Control	ISP2 medium	Resin extract	Medium control
WSS_BLK_Amedia_01.mzXML	Control	A medium	Resin extract	Medium control
WSS_BLK_RAM2_01.mzXML	Control	RAM2 medium	Resin extract	Medium control

Supplementary Table 6. LC-MS/MS raw data files deposited in GNPS MassIVE (accession number MSV000100974).
