# Supplementary material for: Streptomyces produce a diphtheria toxin-like exotoxin that targets insects
Source: Nat Microbiol. 2026 Apr 30;11(5):1271–85. doi: 10.1038/s41564-026-02315-5 (PMC13171429; doi:10.1038/s41564-026-02315-5)
Supplement: Supplementary file 2 — Reporting Summary [file 41564_2026_2315_MOESM2_ESM.pdf]

nature portfolio

Corresponding author(s): Norbert Perrimon, Cameron Currie, Min Dong

Last updated by author(s): 2-25-2026

Reporting Summary

Nature Portfolio wishes to improve the reproducibility of the work that we publish. This form provides structure for consistency and transparency in reporting. For further information on Nature Portfolio policies, see our Editorial Policies and the Editorial Policy Checklist.

Statistics

For all statistical analyses, confirm that the following items are present in the figure legend, table legend, main text, or Methods section.

n/a	Confirmed	
☐	☒	The exact sample size (*n*) for each experimental group/condition, given as a discrete number and unit of measurement
☐	☒	A statement on whether measurements were taken from distinct samples or whether the same sample was measured repeatedly
☒	☐	The statistical test(s) used AND whether they are one- or two-sided<br>*Only common tests should be described solely by name; describe more complex techniques in the Methods section.*
☐	☒	A description of all covariates tested
☒	☐	A description of any assumptions or corrections, such as tests of normality and adjustment for multiple comparisons
☐	☒	A full description of the statistical parameters including central tendency (e.g. means) or other basic estimates (e.g. regression coefficient) AND variation (e.g. standard deviation) or associated estimates of uncertainty (e.g. confidence intervals)
☐	☒	For null hypothesis testing, the test statistic (e.g. *F*, *t*, *r*) with confidence intervals, effect sizes, degrees of freedom and *P* value noted<br>*Give P values as exact values whenever suitable.*
☒	☐	For Bayesian analysis, information on the choice of priors and Markov chain Monte Carlo settings
☒	☐	For hierarchical and complex designs, identification of the appropriate level for tests and full reporting of outcomes
☒	☐	Estimates of effect sizes (e.g. Cohen's *d*, Pearson's *r*), indicating how they were calculated

Our web collection on statistics for biologists contains articles on many of the points above.

Software and code

Policy information about availability of computer code

Data collection: *Provide a description of all commercial, open source and custom code used to collect the data in this study, specifying the version used OR state that no software was used.*

Data analysis: All data were processed using GraphPad Prism version8. Images were processed using imageJ bundled with 64-bit java 1.8.0_172, and Adobe illustrator version2. illumina sequencing data from CRISPR screens were processed using MAGeCK version 0.5.4 or 0.5.9.4.

For manuscripts utilizing custom algorithms or software that are central to the research but not yet described in published literature, software must be made available to editors and reviewers. We strongly encourage code deposition in a community repository (e.g. GitHub). See the Nature Portfolio guidelines for submitting code & software for further information.

Data

Policy information about availability of data

All manuscripts must include a data availability statement. This statement should provide the following information, where applicable:

- Accession codes, unique identifiers, or web links for publicly available datasets
- A description of any restrictions on data availability
- For clinical datasets or third party data, please ensure that the statement adheres to our policy

All biological materials are available upon request from the co-corresponding authors. Crystallographic data statistics are summarized in Table S1. The atomic coordinates and structure factors (PDB code 9QE9) have been deposited in the Protein Data Bank (http://wwpdb.org).

Research involving human participants, their data, or biological material

Policy information about studies with human participants or human data. See also policy information about sex, gender (identity/presentation), and sexual orientation and race, ethnicity and racism.

Reporting on sex and gender	NA
Reporting on race, ethnicity, or other socially relevant groupings	NA
Population characteristics	NA
Recruitment	NA
Ethics oversight	NA

Note that full information on the approval of the study protocol must also be provided in the manuscript.

Field-specific reporting

Please select the one below that is the best fit for your research. If you are not sure, read the appropriate sections before making your selection.

☒ Life sciences ☐ Behavioural & social sciences ☐ Ecological, evolutionary & environmental sciences

For a reference copy of the document with all sections, see nature.com/documents/nr-reporting-summary-flat.pdf

Life sciences study design

All studies must disclose on these points even when the disclosure is negative.

Sample size	For all the analysis based on micrographs, at least three micrographs from three experiments were quantified. No statistical methods were used to pre-determine sample sizes but our sample sizes are similar to those reported in our previous publication (https://www.nature.com/articles/s41586-022-05250-7)<br>For the CRISPR screening experiments,>1000 cells per sgRNA were represented at all the time, a standard number of cell/sgRNA according to previous studies:https://www.nature.com/articles/s41586-022-05250-7
Data exclusions	No data are excluded from analysis
Replication	All the experiments have been replicated at least 3 times.<br>All attempts at replication were successful.
Randomization	This is not relevant to our study because all the experimental groups are treated technically identical. Therefore, we expected little batch to batch variation and did not require randomization.
Blinding	our experiments did not include subjective measurements and therefore did not require blinding

Reporting for specific materials, systems and methods

We require information from authors about some types of materials, experimental systems and methods used in many studies. Here, indicate whether each material, system or method listed is relevant to your study. If you are not sure if a list item applies to your research, read the appropriate section before selecting a response.

Materials & experimental systems

n/a	Involved in the study	
☐	☒	Antibodies
☐	☒	Eukaryotic cell lines
☒	☐	Palaeontology and archaeology
☐	☒	Animals and other organisms
☒	☐	Clinical data
☒	☐	Dual use research of concern
☒	☐	Plants

Methods

n/a	Involved in the study	
☒	☐	ChIP-seq
☒	☐	Flow cytometry
☒	☐	MRI-based neuroimaging

Antibodies

Antibodies used	anti-eEF2 primary antibodies (1:1000,C-9, sc-166415, Santa Cruz)<br>anti-mouse HRP secondary antibodies (1:5000, 31430, Invitrogen)<br>anti-HA antibodies (1:100, 2367S, Cell Signaling Technology)<br>anti-mouse Alexa-568 labelled secondary antibodies (1:500, A11004, invitrogen)
Validation	The anti-eEF2 primary antibody has been used for western blot in many published papers listed in https://www.scbt.com/p/ef-2-antibody-c-9?srsltid=AfmBOopHNOjsip3shXDjhjMGxZhdhdY6kl5Ptv_PvNvitc7R1MYRSXPS<br>The anti-HA antibody has been used for IF in many published papers listed in https://www.cellsignal.com/products/primary-antibodies/ha-tag-6e2-mouse-mab/2367?srsltid=AfmBOooT8Jv0tQaxXKPkdbhRSZ7KUaEfxSb06alOwBUxf50mdi1830Vi

Eukaryotic cell lines

Policy information about cell lines and Sex and Gender in Research

Cell line source(s)	Drosophila melanogaster S2R+, Aedes aegypti Aag-2 and Anopheles coluzzii Sua5B cells were provided by N. Perrimon lab. A subline of S2R+ cells and Sua5B cells expressing SpCas9 and containing an attP integration site, S2R/NPT005/MT-CAS9 (PT5/Cas9) and Sua5B-IE8-Cas9 were described previously and are available at the Drosophila Genomics Resource Center. All cells were grown in Schneider's Media (21720-024, Thermo Fisher Scientific) containing 10% fetal bovine serum (16140-071, Thermo Fisher Scientific) and 1X Penn/Strep (15070063, Thermo Fisher Scientific). To maintain Cas9 transgene, PT5/Cas9 cells were grown in 200 ng/mL Hygromycin (40051, Calbiochem) and Sua5B/Cas9 cells were grown in 500 µg/mL Geneticin (11811031, Thermo Fisher Scientific).<br><br>HeLa (CCL-2), U2OS (HTB-96), J774A.1 (TIB-67), RAW246.7 (TIB-71), CT26 (CRL-2638) and HEK293T (CRL-3216) cells were obtained from ATCC.
Authentication	None of the cell lines used were authenticated
Mycoplasma contamination	The cell lines were not tested for mycoplasma contamination
Commonly misidentified lines<br>(See ICLAC register)	non

Animals and other research organisms

Policy information about studies involving animals; ARRIVE guidelines recommended for reporting animal research, and Sex and Gender in Research

Laboratory animals	v39596 was obtained from Vienna Drosophila Resource Center. Hml-gal4, UAS-GFP and w1118 strain flies were from N. Perrimon lab. Grasshoppers were obtained from Fluker's Fresh Feeder.
Wild animals	No wild animal were used in this study
Reporting on sex	6-10-day-old flies with sex ratio 1:1 were collected and used for the injection experiments.<br>3-day-old D. melanogaster (strain w1118) male or female flies were separately used for the feeding assay.
Field-collected samples	No filed-collected samples were used in this study
Ethics oversight	ethical approval was not required

Note that full information on the approval of the study protocol must also be provided in the manuscript.

Plants

Seed stocks	NA
Novel plant genotypes	NA
Authentication	NA
