# Supplementary figures and images for: Streptomyces produce a diphtheria toxin-like exotoxin that targets insects
Source: Nat Microbiol. 2026 Apr 30;11(5):1271–85. doi: 10.1038/s41564-026-02315-5 (PMC13171429; doi:10.1038/s41564-026-02315-5)

Blots for Extended Data Fig. 4

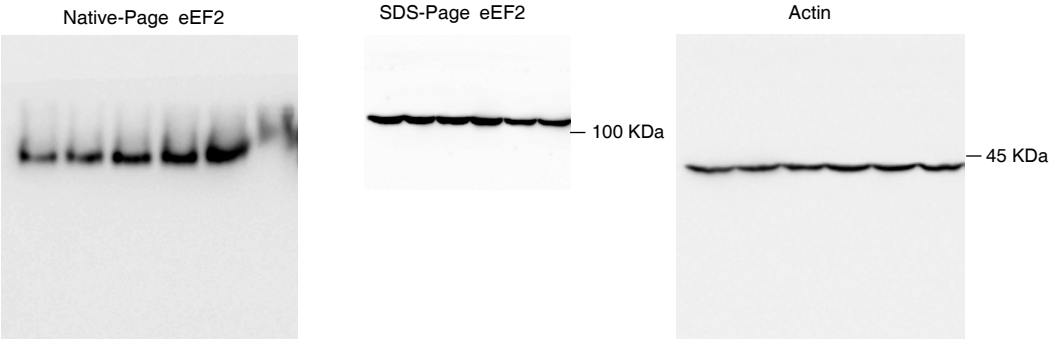

Supplement: Supplementary file 9 — Uncropped blot scans for Extended Data Fig. 4. [file 41564_2026_2315_MOESM9_ESM.pdf]
